# Concentration and geospatial modelling of Health Development Offices’ accessibility for the total and elderly populations in Hungary

**DOI:** 10.1186/s12889-025-22392-1

**Published:** 2025-04-21

**Authors:** Peter Domjan, Viola Angyal, Istvan Vingender

**Affiliations:** 1 Doctoral College, Health Sciences Division, Semmelweis University, Interdisciplinary Applied Health Sciences Program, Vas Street 17, Budapest, 1088 Hungary; 2 Doctoral College, Health Sciences Division, Institute of Digital Health Sciences, Ferenc Square 15, Semmelweis University, Budapest, 1094 Hungary; 3 Faculty of Health Sciences, Department of Social Sciences, Semmelweis University, Vas Street 17, Budapest, 1088 Hungary

**Keywords:** Gerontology, Sociology, Disease prevention, Health geospatial modelling, Ageing, Accessibility, Statistical concentration, Health Development Office

## Abstract

**Background:**

This study examines the availability and national distribution of Health Development Offices (HDOs; *N* = 108) in Hungary, with an emphasis on their role in health prevention for the general and elderly population. HDOs play a crucial role in providing preventive services (nutrition, physical activity, mental hygiene), a significant factor in the health preservation of the elderly. The geographical location and accessibility of these Offices are essential parameters as they influence individual participation willingness.

**Methods:**

Leveraging advanced geospatial modelling techniques with QGIS 3.34.0 and Excel, SPSS software, we mapped the locations of HDOs relative to population centres, employing statistical tools such as the Lorenz Curve and Gini Index, Location Quotient (LQ) Index, and Herfindahl–Hirschman Index. These methods allowed for a nuanced analysis of service concentration and the identification of geographic disparities in service provision. The relationship between the population and the number of HDOs was analysed using Pearson correlation. This spatial and demographic study was based on 2022 data.

**Results:**

The number of HDOs did not indicate significant spatial concentration relative to the population, although the entropy index measured substantial diversity among the counties. Based on the measured LQ Index values, it can be stated that the presence of HDOs is underrepresented in the capital city and several counties. Additionally, our regression analysis indicated that an increase in population size is associated with an increase in the number of HDOs in rural areas; however, this relationship does not hold in Budapest and its surrounding region.

**Conclusion:**

The examination of geocoordinates through scatter plots indicated a broad spectrum of dispersion, and the placement of HDOs on the map revealed a star topology. From the findings of our research, it can be concluded that the Hungarian network of Health Development Offices (*N* = 108) can meet the preventive health needs of both the general and the elderly population. Enhancing the geographical spread of HDOs is crucial for improving the accessibility and effectiveness of disease prevention strategies, especially among Hungary’s aging population, thereby contributing to a more equitable health service landscape.

**Supplementary Information:**

The online version contains supplementary material available at 10.1186/s12889-025-22392-1.

## Introduction

In our study, we aimed to examine the coverage of Health Development Offices (HDOs) in Hungary as providers of disease prevention services among both the general and elderly populations. The HDOs are community-based, publicly funded institutions in Hungary that provide primary prevention services, including health promotion programs, individual health assessments, and education on nutrition, mental health, and physical activity, aiming to improve public health outcomes across all age groups. Our choice of topic was motivated by the increasing importance of disease prevention today, which necessitates an active institutional system and communication [1]. Further effort and financial resources are required to expand the support network for health development. Short-term social rationality understandably finances disease treatment and rehabilitation over prevention, depending on available resources [2]. However, in the long term, a nationwide disease prevention network is necessary to avoid a drastic increase in health financing costs [3, 4]. Further evidence shows that expanding a health prevention network leads to significant long-term cost reductions in health financing and improved population health outcomes [5]. Prevention plays a crucial role in reducing premature deaths and disability, a focus that has gained increasing importance in public health discussions. As Bauer et al. highlighted, the 21st-century approach to health should prioritize eliminating the leading preventable causes of death and disability through comprehensive prevention strategies [6]. Therefore, examining the geographical placement of Health Development Offices is crucial because health development can be defined as part of public health, and if we wish to see improvement in our morbidity and mortality rates by increasing the number of healthy life years, nationwide coverage of health prevention services is essential [7]. Recent studies have shown that healthcare services must be geographically well-placed to ensure equitable access to preventive care, especially in underdeveloped regions [8].

Investigating the evolution of Health Development Offices, it can be said that until 2010, Hungary primarily saw isolated and often independent disease prevention efforts [9]. To improve health-related attitudes and value systems, the first office network was established in 2014, based on the Swiss Model [10]. With the aid of European Union funds, a network of 61 Health Development Offices was established in 2014 [11]. As a further step forward in development, the concept of Health Development and Health Development Offices appeared in the 1997 Act CLIV. of Hungarian law in 2016, prescribing active collaboration with municipalities in local health development [9]. Between 2014 and 2020, with additional European Union funds, the number of Health Development Offices increased to 113. However, the health prevention network can only be most effective if it appears with even coverage and low concentration values [11–13].

The primary goal of establishing the health development network is to positively develop health-related behaviour, including among the elderly population [14]. Rose’s foundational work highlights the importance of addressing not just ‘sick individuals’ but also ‘sick populations,’ which underscores the necessity of population-level interventions like the disease prevention to improve public health outcomes [15]. A crucial aspect of its development is the population’s ability to easily access the nearest Health Development Office from their residence. Despite a high number of Health Development Offices, if the Offices are only accessible to a portion of the population, or if difficult access due to a lack of transportation infrastructure becomes a significant barrier, it’s crucial to establish a well-covered, accessible, and barrier-free health prevention service network [16].

Generally, the prevention programs of Health Development Offices are diverse and can dynamically adapt to the local population’s needs. Their preventive activities fundamentally cover three main areas: nutrition, mental hygiene [17], and physical therapy [11, 18].

HDOs are typically staffed by health professionals, such as public health experts, nurses, physical therapists, mental health professionals, and social workers. In most cases, doctors are not permanently stationed in these offices, but there is collaboration with local general practitioners and specialists when needed (e.g., addiction prevention programs involve addiction specialists).

The primary task of HDOs is to promote primary prevention and health development through individual and group health promotion programs, which they organize themselves. They also conduct individual health assessments. Additionally, they use their communication channels to advertise national screening programs, highlighting the importance of secondary prevention.

HDOs are organized and overseen by the National Directorate General for Hospitals (OKFO) in Hungary. The OKFO is responsible for setting the operational framework of the HDOs and ensuring the coordination of their programs. They work in collaboration with local municipalities and healthcare providers to tailor services to regional health needs. The HDOs are aligned with Hungary’s national health strategy, and their establishment was carried out in several stages with funding from European Union grants. The EU grant for establishing the offices primarily focused on economically disadvantaged areas and vulnerable regions (2014–2020). Today, local municipalities can also initiate the opening of new HDOs at their own expense. However, annual funding for their operation can be applied for from the Hungarian state budget. As the initial grants have been exhausted, their operation is now maintained through public funds. The ownership background of HDO service providers typically consists of local municipalities or outpatient care institutions. Based on the local population composition, the Health Development Offices provide health prevention services for both the young and active, as well as the elderly age groups, potentially serving as a useful gerontological and health prevention location specifically for those over 64. For the elderly population, the proximity of the Offices and barrier-free accessibility and utilization of transportation infrastructure is especially important.

Health development faces several societal barriers, including issues related to socialization, health-related attitudes, lack of information, and environmental factors, which are general problem [19]. Various regions have been shown to influence societal barriers, including health attitudes and environmental factors, which affect health promotion efforts and the uptake of preventive services [20]. It’s essential that the Hungarian population internalizes the knowledge that impacts health-related thinking and actions for health, even in old age [21]. This requirement necessitates low concentration and even coverage from the side of the Health Development Office network.

Is the coverage of Health Development Offices operating in Hungary proportional to the population of the counties, or does it show a high concentration among certain areas according to some principle? Answering this question requires geographic information system analysis with statistical concentration measurement, which is a prerequisite for a list of Health Development Offices’ locations for further analyses based on various health indicators and transportation infrastructure.

Our research uses geospatial modelling and the adaptation of statistical concentration, we aimed to describe a methodology through a specific public health example that can be applied in further similar research studies. Our geospatial analysis applied statistical concentration and coverage models to assess the accessibility of Health Development Offices in Hungary. This methodology aligns with recent studies that evaluate healthcare accessibility through multi-criteria decision analysis and GIS-based approaches [22].

We hypothesize that the spatial distribution of Health Development Offices (HDOs) in Hungary is not proportional to the county population size and shows significant geographical disparities, particularly in economically disadvantaged regions. For the population, these spatial disparities are especially important in terms of access to primary prevention services.

## Methods

### Study design and data collection

Our cross-sectional and quantitative study sought to explore the spatial patterns formed by HDOs across Hungary and their territorial concentration among the general population as well as among the elderly population aged 64 and over.

Initially, our objective was to create an Excel database containing the contact information (postal code, municipality, precise address) and names of Health Development Offices. The addresses for these offices were obtained from the National Public Health Center's website (https://www.nnk.gov.hu/index.php/efi) (107 addresses) [23], to which we also added the contact information of the Health Development Centre at Semmelweis University. Utilizing the addresses of 108 Health Development Offices, we generated geo coordinates with the help of Google Maps online (https://maps.google.com/) [24], following the WGS 84 standard for extracting latitude and longitude values. These geo-coordinate values were then recorded in the mentioned Excel database. These offices were still operational in 2022.

For spatial representation, we used QGIS (Quantum Geographic Information System) version 3.34, an open-source software that includes the freely accessible world map provided by the OpenStreetMap Foundation, encompassing a complete map of Hungary [25]. The source from OpenStreetMap included the full Hungarian administrative map [26], detailing the boundaries of municipalities, counties, and districts, thus facilitating the creation of our spatial database for the study.

The number of HDOs and the county population (total and elderly) were also recorded in SPSS 29 and MS Excel, which we used for Pearson correlation and linear regression analysis, and goodness-of-fit testing.

Utilizing our database, which contained addresses and associated geo coordinates, we imported the data into the QGIS geographic information system. This allowed us to visualize the geographical distribution of Offices across the Hungarian administrative map, based on the latitude and longitude coordinates [27]. The QGIS program was used to visualize the HDO offices using geocoordinates, with the county boundaries treated as a separate layer. We analysed the service areas of the HDO offices by applying 5 buffer zones of 1.5 km each and created a heatmap using Kernel Density Estimation to examine the spatial distribution, with a radius of 7.5 km.

In addition to the geospatial representation, our analysis was mainly based on the examination of the statistical concentration of the county population size and the Health Development Offices. County-level population data by age group were obtained from the website of the Hungarian Central Statistical Office [28], which provides the total population for each county in 2022 and the population over 64 years of age. A further prerequisite for our statistical investigations was the aggregation of the number of operational Health Development Offices per county based on their available addresses for 2022, which was the basis for our concentration calculations.

In our research, we examined the spatial distribution of HDOs from multiple dimensions using the following statistical indicators. The combination of the Lorenz curve, Gini index, Location Quotient, Herfindahl–Hirschman Index, and entropy index allows for a multidimensional analysis of HDO distribution. While the Lorenz Curve and Gini Index measure general inequality in the spatial distribution, the LQ provides insights into regional concentrations. The HHI further quantifies concentration, and HHI helped validate the findings from the Lorenz Curve, confirming that the distribution of HDOs. Our research is enhanced by the application of information-theoretic Shannon Entropy (H), which is referred to as entropy throughout our manuscript. Together, these tools enable a nuanced understanding of spatial disparities in the distribution of HDOs, offering a more complete picture of accessibility and equity.

The statistical calculations were performed using a standardized database, and the detailed computational results (dataset), along with a brief statistical description, were uploaded to the CERN scientific data repository, Zenodo, on 23rd September 2024. This upload aims to ensure transparency and the reproducibility of our calculations [29].

### Analysis with standardized data

Due to the territorial comparison involving Health Development Offices, it was necessary to apply the method of statistical standardization [30, 31]. This process involved using the available county population data and the frequency data of Health Development Offices per county (f(x)) to calculate the indicator of the number of Health Development Offices per 100,000 inhabitants.

($\frac{Number\; of\; Health\; Development\; Offices\; at\; the\; county\; level}{Population\; of\; county} x 100\; 000$) Our statistical analysis focused on the total population of each county and the population aged over 64. The purpose of standardization was to make counties with differing population sizes comparable in terms of the territorial distribution of operational Health Development Offices.

### Used statistical tools

*Descriptive statistics* (mean; minimum value, maximum value; standard deviation, coefficient of variation) [32]. Our statistical calculations required the measurement of these indicators, providing basic information for further analysis of our examined variables.

#### Lorenz Curve with standardized data

The use of the Lorenz Curve required statistical standardization based on the differing population sizes of counties. This curve was designed to assess the disparities in the allocation of Health Development Offices across counties, considering the proportions of the general and elderly populations.

#### The Gini Index (G) facilitated the validation of our Lorenz Curve [33]

$$G=\frac{1}{2{n}^{2}\overline{x}}{\Sigma }_{i=1}^{n}{\Sigma }_{j=1}^{n}|{x}_{i}-{x}_{j}|$$

n: number of Hungarian counties

x: number of HDOs per 100,000 population in the examined county

#### Location Quotient (LQ) Index

The LQ index allowed for the measurement of the concentration of Health Development Offices by county, comparing these figures to national data. A value greater than 1 indicated a higher concentration in the county relative to national figures. This indicator did not require statistical standardization based on population size.

##### (LQ) index [34]

$$LQ=\frac{\frac{Number\; of\; HDOs\; in\; an\; examined\; county}{HDOs\; number\; in\; Hungry}}{\frac{The\; population\; of\; the\; examined\; county}{Total\; population\; in\; Hungary}}$$

#### Herfindahl–Hirschman Index (HHI)

The basis for using the HHI Index was the relative frequency value of Health Development Offices per county.

##### (HHI) index

$$HHI= \sum\nolimits_{i=1}^{n}{(Si)}^{2}$$

n: number of counties, ${S}_{i}$: Health Office distribution ratio [35]

#### Entropy index (H)

The diversity and uniformity of distribution across the national sample were measured using the entropy index, which indicates the territorial diversification of the offices. The application of a base- 10 logarithm facilitates easier interpretation of the results, commonly used in health and sociological research.

##### Entropy index [36]

$$H=-\sum_{i=1}^{n}{p}_{i}{\times log}_{10}({p}_{i})$$

n: number of counties

${p}_{i}$ relative frequency of Health Development Offices in a selected country [36]

#### Regression analysis and Pearson correlation calculation

In our research, we used a univariate linear correlation equation to illustrate the relationship between the population size of counties and the number of Health Development Offices (HDOs). The scatter plot visualization was created by interpolating the available data (x: population of the county; y: number of Health Development Offices in a given county). While this method allowed us to visually depict the relationship, our primary objective was not to predict future outcomes but rather to clarify the current distribution patterns.

To evaluate the fit of the regression model, we conducted an analysis of variance (ANOVA), which allowed us to examine, with 95% confidence, whether the predicted changes in the dependent variable (number of Health Development Offices) were statistically significant.

We further assessed the model fit by analysing the residuals. Residuals, which represent the differences between observed and predicted values, are crucial in evaluating the accuracy of the model. Specifically, analysing the residuals helps identify any potential patterns or deviations that could suggest the model does not adequately meet the assumptions of linear regression, such as normality and homoscedasticity.

In our study, we also examined the coefficient of determination (R-squared), a key indicator for regression models. The R-squared value indicates the proportion of the variance in the dependent variable that is explained by the independent variable(s). This metric provides critical information about how well the model fits the data.

It is important to note that our intent was not to forecast future trends using the linear regression model. Instead, we used it to visually support the interpretation of Pearson’s correlation coefficient, demonstrating the relationship between the two variables. Our findings were further validated using both the correlation coefficient and the coefficient of determination (R-squared), which helped us assess how much variability in the number of Health Development Offices (HDOs) (y) could be explained by the population size (x) in the counties. This approach allowed us to better understand the present relationship without attempting to predict future changes [30, 36].

## Results

### Location analysis of Health Development Offices

**Fig. 1:**
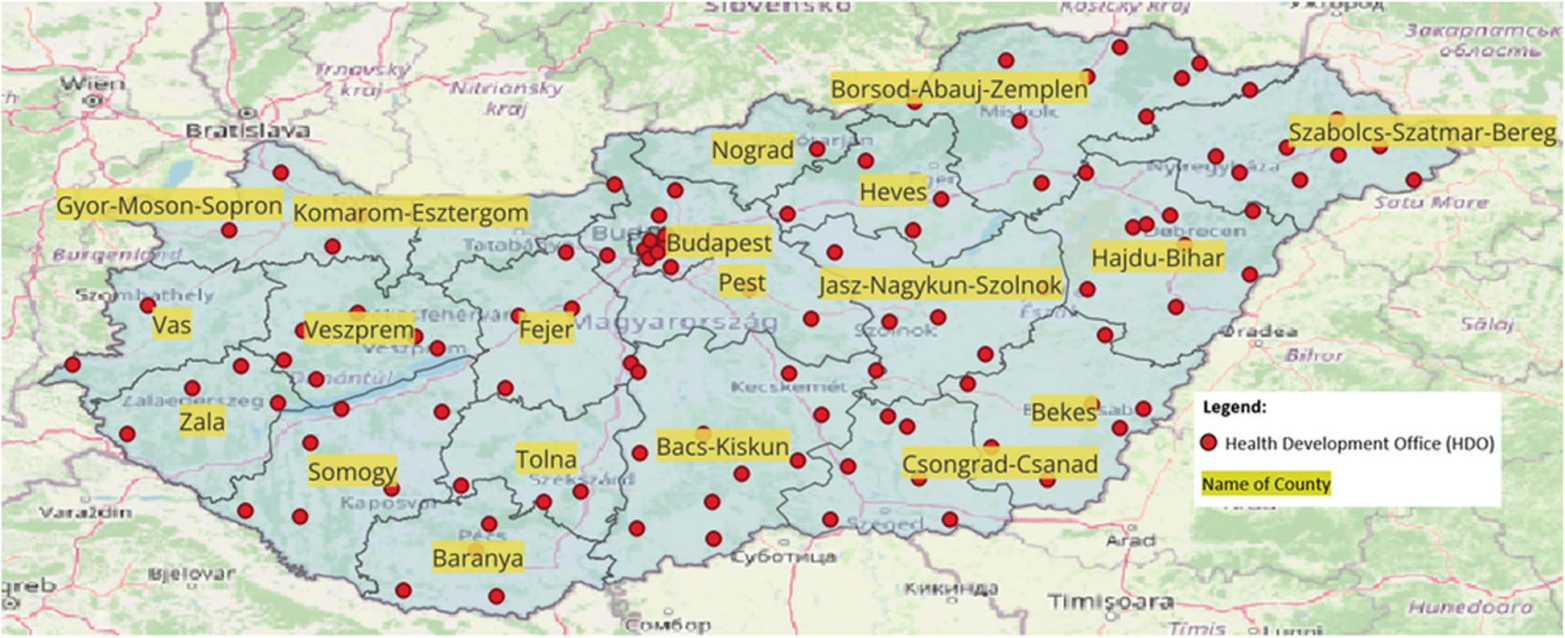
Pattern of spatial placement of Health Development Offices in 2022

Our spatial modelling covered 19 counties and the administrative area of Budapest, encompassing a total of 108 Health Development Offices. Our analysis revealed a strong diversification among the counties in terms of the number of operational Health Development Offices (see Table 1). Including Budapest, the average number of Health Development Offices per county was 5.68, with a range from 0 to 11 Health Development Offices in the examined territorial units. The spatial placement patterns of the Health Development Offices indicated that, with the exception of Komarom-Esztergom County, at least one Health Development Office operates in every county. Based on the spatial analysis, we observed that Health Development Offices are unevenly distributed across Hungary, with 108 offices providing coverage to various regions, though with noticeable disparities in accessibility. The examination of geocoordinates through scatter plots, based on latitude and longitude, indicated a broad spectrum of dispersion, and the placement of Health Development Offices on the map revealed a pattern radiating from a central hub, suggesting a star topology (see Fig. 1).

**Table 1:**
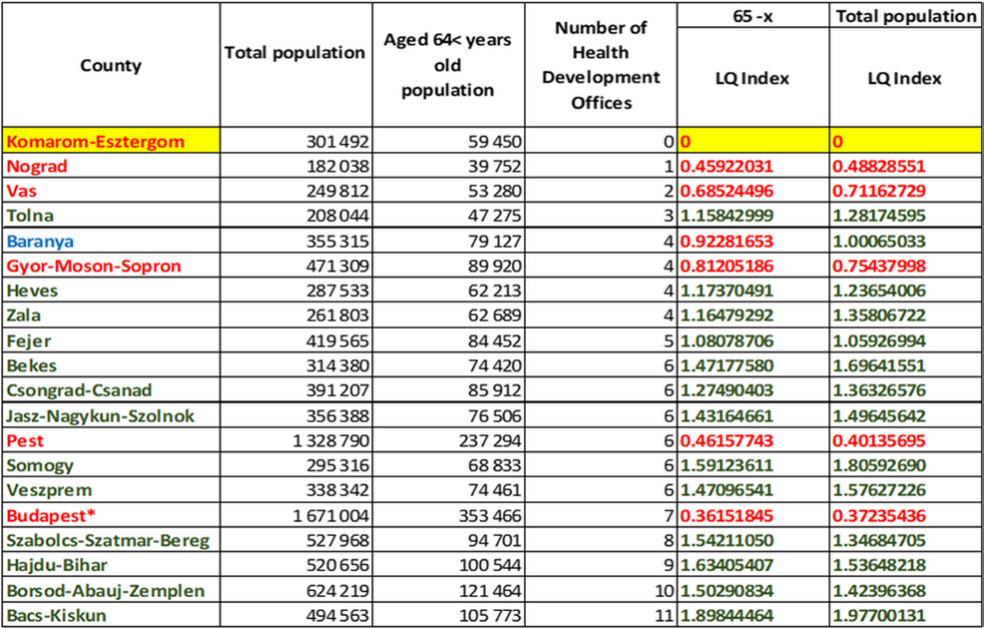
Location Quotient Index of Health Development Offices based on the population of counties

Beyond the geocoordinates of the HDOs, we created a heatmap of Hungary’s HDO offices using the geocoordinates of the HDOs and a Kernel density estimation function, which shows the counties with a higher number of HDOs. The pixel size was set to 0.01 during the Kernel density estimation, and the calculation radius was 7.5 km.

**Fig. 2:**
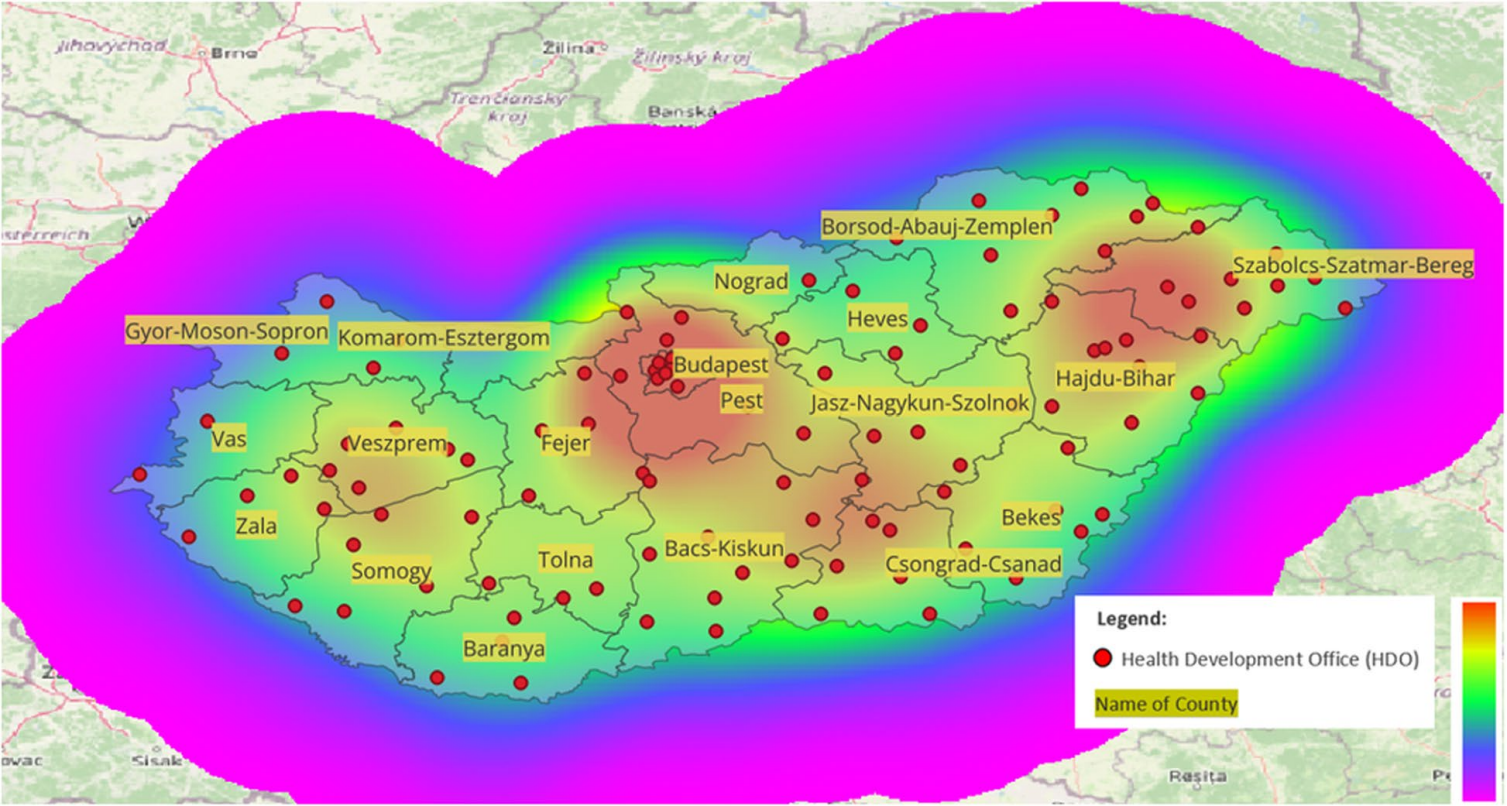
Kernel density heatmap of Health Development Offices (HDOs) in Hungary by county

As shown in Fig. 2, Budapest and its surroundings, as well as Hajdu-Bihar and Szabolcs-Szatmár-Bereg Counties, have the highest number of HDO offices. There is also a higher concentration near certain county borders; however, it is important to examine the catchment areas these offices can serve.

**Fig. 3:**
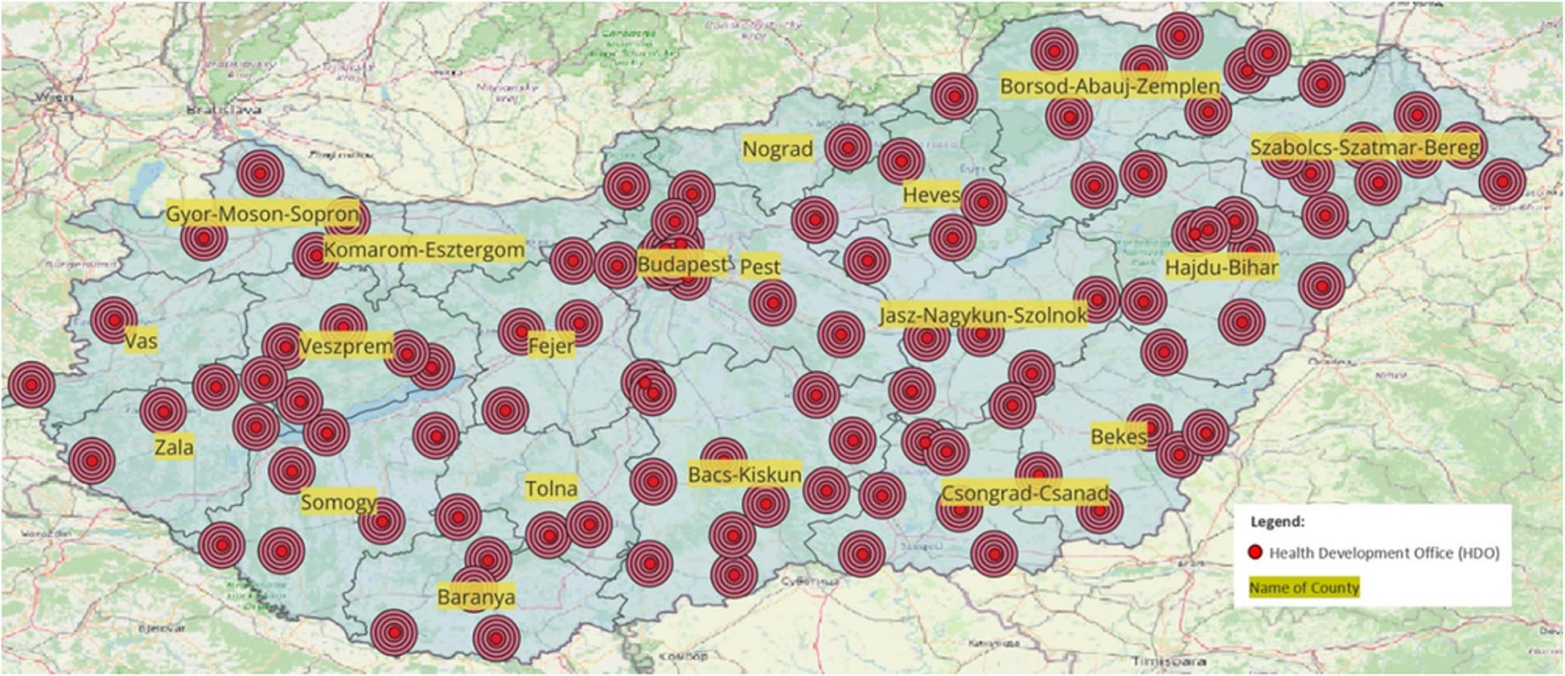
Distribution of HDOs in Hungary by county with 1.5 km Buffer Zones (5 Zones/HDO)

The map (Fig. 3) illustrates the spatial distribution of Health Development Offices (HDOs) across Hungary with a buffer analysis that highlights five concentric buffer zones, each with a radius of 1.5 kms, around each office. These five buffer zones represent areas within which primary prevention services are most readily accessible, assuming that populations living within these zones can easily reach an HDO without significant transportation barriers.

This 1.5 km distance per buffer zone, with a total of five zones, is an arbitrary measure used to visualize potential areas of service. However, transportation infrastructure, population density, and geographical challenges may influence real accessibility. This visualization allows us to assess potential service overlaps and gaps in coverage across different counties, revealing regions that may benefit from additional HDOs to ensure equitable access to disease prevention services.

The reason for the mentioned spatial arrangement is partly due to the pattern of Health Development Offices aligning with the population data of the various counties. The emergence of this spatial structure was influenced by the fact that 31.24% of the Hungarian population is concentrated in Budapest and its surrounding county (Pest) [28].

To map out county-level differences, our analysis performed a Location Quotient (LQ) calculation to determine which counties are underrepresented relative to the national average.

Based on Table 1, it is evident that Health Development Offices are less concentrated in Nograd, Vas, Gyor-Moson-Sopron Counties, as well as in Budapest and Pest County. The Location Quotient (LQ) Index value is less than 1 indicating that the concentration of Health Development Offices is lower than the national average. It is also noteworthy that as of January 2024, there are no operating Health Development Offices in Komarom-Esztergom County, which could be attributed to the presumed passivity of the relevant municipalities and healthcare stakeholders or the lack of infrastructure necessary for establishing an Office. When examining the LQ Indexes for the total population and the elderly population, a similar pattern emerges in terms of location concentration, except Baranya County becoming underrepresented in terms of the elderly population size and the number of Health Development Offices.

### Statistical concentration analysis

In addition to the Location Quotient Index, we used the Lorenz Curve to examine the concentration resulting from the population of the counties and the number of Health Development Offices located within them, both for the total and the elderly population, from which conclusions about the Offices’ territorial distribution can be drawn.

The Lorenz Curve, expressing the degree of concentration, contrasts the cumulative increase in population size with the growth in the number of Health Development Offices, the latter in ascending order serving as the basis for the analysis. In the Cartesian coordinate system, a 45-degree reference line symbolizes the absence of concentration, representing perfect distribution, and the area between this line and the Lorenz Curve indicates the magnitude of concentration. In relation to this area, the Gini Coefficient can be interpreted, which can take on values between 0 and 1, where 0 represents perfect equality and 1 represents complete inequality in terms of the variables examined [33].

The comparison required statistical standardization, hence the study was based on the number of Health Development Offices per 100,000 population.

**Fig. 4:**
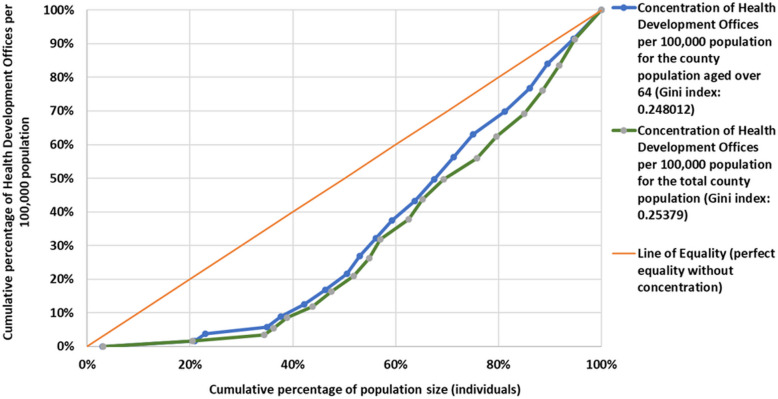
Lorenz curve of Health Development Offices per 100,000 people based on county population

Figure 4 shows the concentration of Health Development Offices as a function of the total population and the over- 64 population of the county, using the Lorenz Curve.

**Fig. 5:**
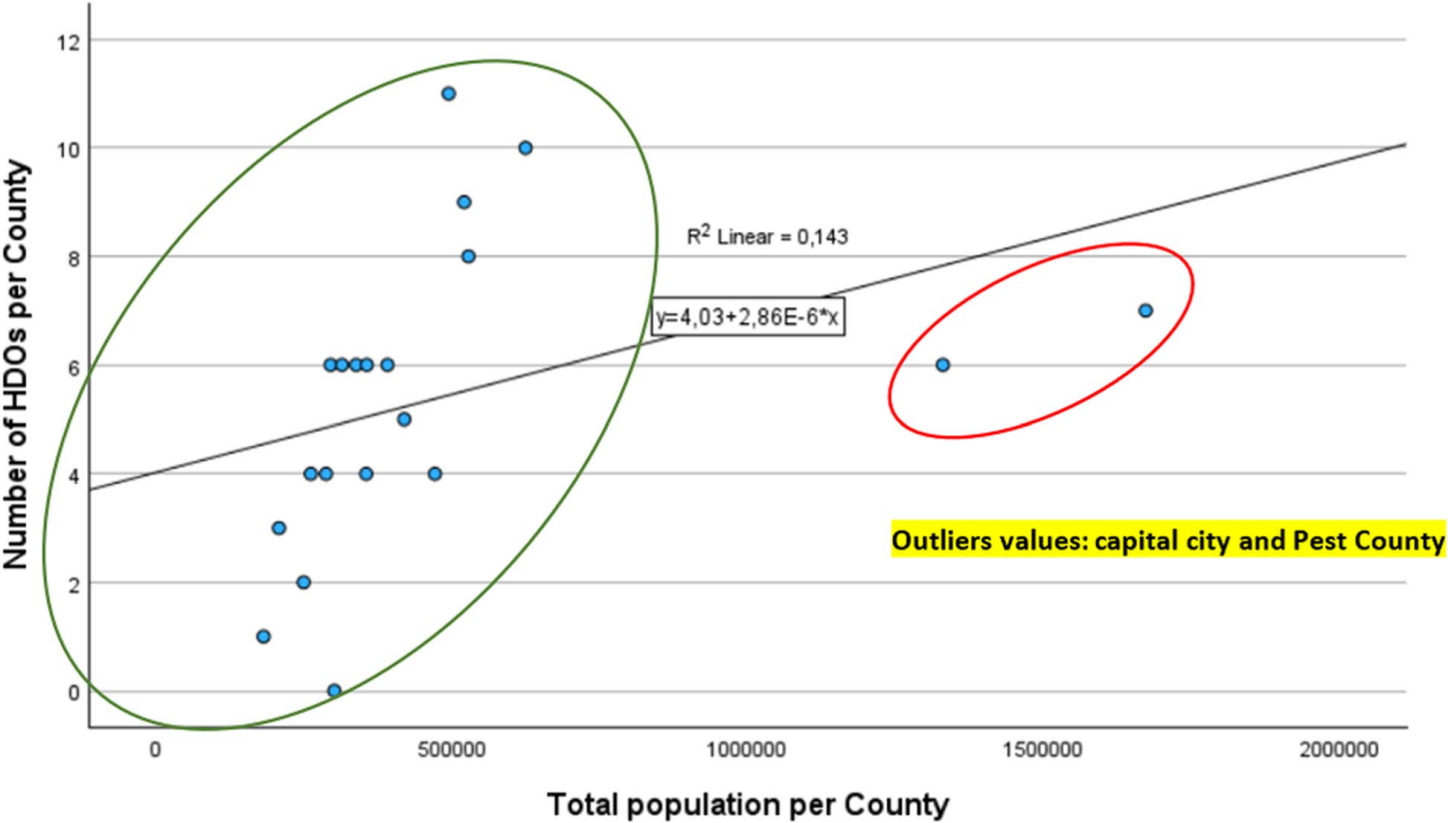
Stochastic relationship between total county population and the number of Health Development Offices

**Fig. 6:**
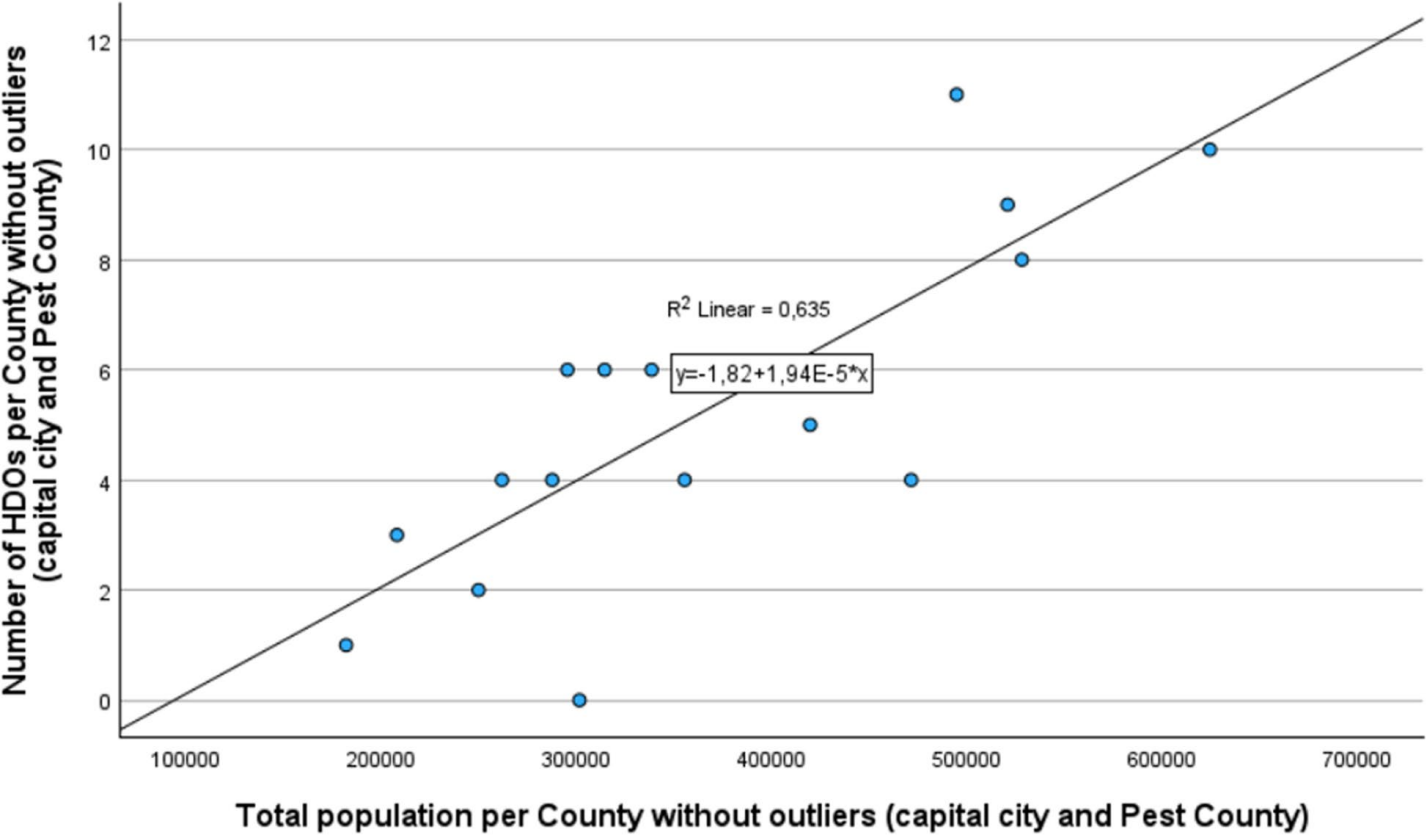
Stochastic relationship between total county population and the number of HDOs without outliers value

At higher concentrations, the Lorenz Curve converges towards the lower right corner. Based on the shape of the Lorenz curve for both the total population and the (65-x) population, it can be concluded that the concentration of Health Development Offices in Hungary is low. For both the total population and the older generation, a Gini coefficient around 0.25 indicates a relatively low level of inequality in the distribution of the number of Health Development Offices across different counties. Therefore, the distribution of Health Development Offices is relatively even, though variations exist among the counties, caused by the underrepresented counties as measured by the Location Quotient Index (Table 1).

We validated our measurement results with the Herfindahl–Hirschman Index (HHI), aimed at checking our findings. The indicator can take values between 0 and 1, with the distribution becoming more uniform as it approaches zero.

In our research, the HHI Index helps to understand the extent to which Health Development Offices are concentrated in each county. Based on the number of Health Development Offices in the counties, the Herfindahl–Hirschman Index value was 0.063, indicating a low concentration, thus supporting our previous measurement results. The value measured in our study signifies a low concentration of Health Development Offices in the examined counties, assuming an even distribution and confirming the values measured by the Lorenz Curve. The indicator supports that the Offices are widely distributed among the counties and are not concentrated in a few.

### Measure of diversification

With the entropy index, we aimed to measure the diversity of the standardized indicator value of Health Development Offices per 100,000 people. This statistical indicator is also used in thermodynamics instead of public health research, but it is capable of expressing the degree of diversification or uncertainty, suitable for further evaluation of our study [36]. The entropy index, calculated based on the number of Health Development Offices per 100,000 inhabitants across counties, was 1.2431. This value signifies a considerable variation in the distribution of Health Development Offices relative to the total population, despite previous measures indicating a low statistical concentration that suggested uniform national distribution. From the maximum value of entropy, ${log}_{10}\left(20\right)=1.30103$, it follows that the entropy value is high, which can be explained by the variations among the counties in terms of standardized values.

Regarding the aging demographic, the entropy index value for the number of Health Development Offices per 100,000 elderly individuals (65 -x) was calculated at 1.2454. This figure is attributed to variations in the Location Quotient Indexes across counties, as detailed in Table 1. The distribution of Health Development Offices indicates an even distribution at the county level for both the aging and the total population, yet the number of Health Development Offices per 100,000 elderly was changeable among the counties, creating higher diversity in the distribution. Based on the number of Health Development Offices per 100,000 people in each county, the entropy index value for the total population was 1.2431, indicating high variability in Health Development Offices concerning the total population, despite the low statistical concentration measured earlier suggesting national coverage.

From the statistical results, it can be concluded that the accessibility of Health Development Offices varies across counties for both the total and elderly populations, but does not show significant concentration or isolation in the representation of the HDOs, as also supported by the low Gini Coefficient value.

### Examining the relationship between population and Health Development Offices (HDOs)

Analysing the stochastic relationship between county population and the number of Health Development Offices [23, 28], it is an expected requirement that the number of operating Health Development Offices should increase with population growth. For this task, we applied Pearson correlation and linear regression calculations.

If we want to conduct a retrospective analysis between the total population of the counties and the number of HDOs per county, a weak Pearson correlation of 0.378 is observed. A similar Pearson correlation value of 0.375 is found for the elderly population over 64 years old. In our research, the relationship between the independent variable (total population per county) and the dependent variable (number of HDOs per county) is presented using a regression line, without the intention of future data adaptation (Fig. 5).

If we examine the stochastic relationship between the county population and HDOs, we find two outlier values (the capital city and Pest County), where the deviation can be explained by the fact that the establishment of HDOs in Hungary between 2014 and 2020 was financed by EU tenders, which, apart from the economically developed capital and Pest County, focused primarily on disadvantaged and public health-threatened areas. Due to the outlier values, the fit of the regression resulted in a low R-squared value, indicating that the population (independent variable) explains only 14.3% of the variance of the dependent variable. The fit of the model is weak and not significant (*p* = 0.101; F: 2.992, residual value: 129.306) with high residual values. Our analysis was performed using SPSS 29.0.1.0. During the fit analysis, a cubic regression function provided the best fit; however, since our research aimed to analyse past data, we proceeded with the examination of the data after removing the outlier values. Without the outlier values from the capital and Pest County, where the HDOs are underrepresented in relation to the population size, we obtained a well-fitting regression line and a strong Pearson correlation value (0.797) for the total population. For the elderly population, the Pearson correlation coefficient was even stronger, showing a value of 0.868, indicating a significant relationship between the dependent and independent variables in both cases (Fig. 6).

Based on the cleaned data (excluding the capital and Pest County), the fit analysis resulted in a stronger determination coefficient (R-squared) of 0.635. This means that, in rural areas, where the EU tenders were focused, the independent variable (population size) influenced the variance of the dependent variable (number of HDOs) by 63.5%. The strength of the trend line was also supported by the fit analysis (*p* < 0.001, F: 27.808, residual value: 53.912). Overall, based on the residual values, the linear model shows a better fit compared to when the outlier values were included in the analysis. Alongside the total population per county, we also performed a linear regression analysis for the elderly population over 64 years old at the county level, with a determination coefficient of 0.141, which increased to 0.753 after removing the outlier values (capital city and Budapest), showing a strong fit of the function (*p* < 0.001; F: 48.673, residual value: 36.519). In summary, it can be concluded that in rural areas, excluding the capital and Pest County, the establishment of HDOs (their number) correlates with the population size. However, due to the stochastic relationship, this fit does not lead to a functional relationship, as there are counties where HDOs are underrepresented compared to the national average.

The graph shows that without the outlier values, in rural areas, where the EU tenders were primarily focused, a strong Pearson correlation can be found between the county population size and the number of HDOs, which is also supported by the regression analysis without future extrapolation of the data.

## Discussion

In regional-based modelling, it is important not only to examine the accessibility of each county based on raw numbers but also to perform territorial modelling aimed at exploring the current situation, because regional disparities in access to healthcare services are often influenced by the availability of healthcare resources [37]. Indicators measuring concentration, with their specific characteristics, can appropriately express the population-proportional accessibility of various healthcare and health prevention services. A novelty of our research is that, in addition to classical indicators, we also examined the informational content of the probability distribution of the HDOs’ locations (using the histogram method) with the entropy based on the logarithm of 10. The advantage of the entropy, besides examining diversity, is that it also expresses the uncertainty of the system (network), which in our case is identified with the dispersion of HDOs. The Gini Index and the Lorenz Curve are not only applicable to the distribution of income and wealth in society, but our research highlights that their classification using a histogram method based on geographical regions provides excellent information from a public health perspective on the distribution and concentration of HDOs.

In this context, a low Gini Index value combined with a high entropy value suggests a more even and widespread distribution of HDOs, indicating that the network achieves good national coverage without significant concentration in only a few areas.

The territorial distribution of HDOs is of paramount importance for both the elderly and the total population due to their role in implementing primary prevention and promoting secondary prevention. Our distribution data provide valuable assistance to decision-makers in the planning of public health policies. Unfortunately, health promotion is often subordinated to some primary healthcare or public health institution and rarely appears as an independent entity. In most countries, healthcare functions as “sick care,” and the healthcare system is rarely visited by asymptomatic (at-risk) individuals for preventive health purposes. In developing countries with low GDP per capita, health promotion is focused on reducing infectious diseases. It is a fact that developed countries face different health issues, where many chronic diseases caused by modern civilization can be prevented, largely depending on individuals’ health-conscious behaviour [5].

From my geospatial modelling, which focused on uncovering the current situation, it can be stated that the establishment of HDOs in Hungary was made possible through EU funds, with their maintenance now financed by the central budget due to the expiration of previous grants (2014–2020). The geospatial data suggest that most offices are located in Budapest and Pest County due to the star topology. However, when considering population size, a much more nuanced picture emerges, revealing the underrepresentation of the capital city and Pest County. The practical benefit of examining the distribution is that it provides a methodology for public health planners, especially those involved in health promotion. A holistic and multidimensional approach ensures that territorial data provide insights into the availability of HDOs. The spatial accessibility of primary care remains a complex issue, with various methods used to measure and assess this aspect, each presenting unique challenges [5]. Without a proper network and measurement tools, the effectiveness of HDOs may be low, particularly among the elderly population, despite societal will [2]. As Guagliardo highlights, spatial accessibility remains a core challenge in the planning and delivery of healthcare services, especially in rural areas, where disparities in health services are often the greatest [5].

If we further examine the population’s age composition, it can be stated that 21% of the population is over 64 years old, which, with an urn-shaped age distribution, indicates an aging society whose economic impact will become increasingly significant in the future in Hungary [38]. For these reasons, the spatial placement and accessibility of Health Development Offices are important factors for the elderly population as well, if there is a desire to increase the number of individuals utilizing health prevention services. The mentioned aging process can be observed in most countries, requiring active social intervention in the field of disease promotion [12].

Therefore, a disease prevention service can achieve long-term results if the network of Health Development Offices has national coverage and is easily accessible to the population, especially the elderly [39]. Besides spatial placement, however, factors influencing service uptake, including transportation options and other sociological health factors, should not be overlooked [40, 41]. In addition to population distribution, healthcare resources, and transportation infrastructure are critical factors influencing the accessibility of health services, as seen in international studies [42]. In particular, Luo and Wang demonstrated that spatial accessibility models are effective in identifying regions with inadequate healthcare coverage, providing a useful framework for understanding geographic disparities in service availability [43, 44].

The availability of transportation infrastructure can significantly improve, or its absence can worsen, the absorption of interested parties from the Office’s catchment area. However, access to health care is not solely dependent on spatial factors; non-spatial factors such as socioeconomic status and demographic characteristics also play a crucial role in determining healthcare accessibility [45]. Therefore, the health value attitudes and knowledge of those living in the area of a Health Development Office can also vary significantly, affecting both the establishment and utilization of the Office [46]. It must not be forgotten that the purpose of Health Development Offices is to improve morbidity indicators that lead to leading causes of death.

It is evident that counties with higher GDP per capita generally have a lower number of Health Development Offices (HDOs) per capita. This trend warrants further investigation to understand the factors influencing the distribution of HDOs, including the criteria used for grant applications that supported their establishment in different regions. The allocation of EU grant resources was primarily aimed at supporting the development of Health Development Offices in regions that were more vulnerable from a public health perspective — those with lower GDP per capita and worse morbidity and mortality indicators. This approach was intended to enhance health promotion efforts in the most disadvantaged and high-risk areas [46]. Significant progress can be made in reducing health risks for both the elderly and the active population through improvements in nutrition, physical activity, and mental health, all of which are essential at every stage of life [47].

The data modelling leads to the conclusion that the EU grant calls appropriately focused on vulnerable areas, as those Hungarian counties with poorer morbidity and mortality indicators have relatively more Health Development Offices (HDOs) compared to the national average. This confirms that the HDO network significantly targeted at-risk counties. According to the Hungarian Central Statistical Office, Bekes County, Borsod-Abauj-Zemplen County, and Szabolcs-Szatmar-Bereg County show exceptionally high mortality rates, particularly due to the prevalence of cardiovascular and cancer-related diseases. Bekés County operates 6 HDOs, a relatively high number given the lower population size (LQ index 1.471). Borsod-Abauj-Zemplen County has 10 HDOs, which represents an appropriate distribution relative to the population (LQ index 1.502). Szabolcs-Szatmar-Bereg County has 8 HDOs, also with a high LQ index (1.542). These vulnerable counties also have unfavorable morbidity data, making the development of HDOs in these areas justified from a public health perspective, especially for influencing population health behaviour.

At the same time, Budapest and Pest County are underrepresented in terms of the number of HDOs relative to their large population. However, most chronic diseases related to modern civilization in these regions are also lifestyle-related, which could be significantly reduced through the improvement of primary prevention. Despite the high GDP per capita and low unemployment rate, the sedentary lifestyle, unhealthy diet, high stress levels, and harmful habits (alcohol consumption and smoking) [48] justify the need for further HDO development in the capital and surrounding areas.

From the degree of concentration, it can be concluded, that despite inequalities, the network of Health Development Offices is suitable for serving the needs of the total and elderly populations. However, coverage is not yet complete, and the location concentration also highlights that there are areas in need of network development, as well as considering capacity development of existing providers in light of demands. Health Development Offices work with similar infrastructure and human resources regardless of the size of the affected district, while significant differences exist between their territorial service areas.

Based on the descriptions, we must see that numerous factors influence the formation of the existing network of Health Development Offices [49]. The star topology of the Health Development Office is partly explainable by the population’s territorial distribution, with transportation, communication, and infrastructure factors also cited as further explanatory reasons.

Central organizational processes, with efficiency and effectiveness in mind, also facilitated the formation of the star topology; however, based on the county differences in the number of Health Development Offices per 100,000 population, it’s evident that besides the mentioned factors, numerous factors influence the spatial placement of Health Development Offices. It’s not coincidental that the capital and its immediate surroundings became underweight based on Location Quotient Indexes.

As Kornyicki [11] revealed, those European Union funds and grants that established the Health Development Offices contributed a lot of targeted objectives such as EU grants focused on improving individual health behaviours, particularly in high-risk areas, and prioritized underdeveloped regions to positively influence the population’s health status. Public health efforts could be strengthened through a systematic approach, ensuring the high quality and consistency of preventive services. Health development should be approached with an integrated perspective, enhancing the role of general practitioners as “gatekeepers.” Furthermore, cooperation between healthcare providers and local social and economic stakeholders needs to be encouraged. The primary goal of establishing HDOs was to improve morbidity and mortality indicators by emphasizing primary and secondary prevention.

An additional important aspect of the establishment of Health Development Offices was that they operate integrally and play a significant role in the implementation of the region’s health development strategy. This function represents an active link, a bridge between the region’s health service providers, the local government, and civil organizations, which is critically important for preserving the health of the elderly, according to Molnár and colleagues and VG Janson & Elisabeth [50, 51].

Facilitated by EU grant funding, 2014 saw the establishment of 20 Health Development Offices in the most socioeconomically disadvantaged districts and an additional 18 in districts categorized as disadvantaged, out of a total of 61 offices [11]. It is an important fact because Socioeconomic status has been shown to significantly affect access to healthcare services [52, 53]. The data shows that the first health development offices served to catch up with underdeveloped areas to improve positive health attitudes and health preservation. Approaching from a health sociology perspective, we can expect significantly worse morbidity and mortality indicators in underdeveloped areas, which can be attributed to socialization, social and geographical environment, and individual values, warranting increased social attention [10].

After 2014, Health Development Offices inaugurated under the EFOP and VEKOP programs expanded their roles to include mental hygiene and mental health services, marking a notable advancement in the field [47]. Remarkable, it was the VEKOP grants that facilitated the inclusion of the capital city into the Health Development Offices network, thereby playing a crucial role in establishing a star topology in the distribution of these services where the population was a crucial influence factor.

The last major phase of establishing Health Development Offices (HDOs) in the capital city and Pest County was completed in 2020, raising the question of how they have influenced morbidity rates. From the morbidity and mortality data available on the Hungarian Central Statistics website, we can conclude that counties with the worst outcomes, such as Bekes, Borsod-Abauj-Zemplen, and Szabolcs-Szatmar-Bereg, have not shown significant improvement in mortality rates since 2012. In these counties, the proportion of deaths from cardiovascular and cancer-related diseases remains high. Unfortunately, in Bekes County, the mortality rate due to cardiovascular diseases increased from 782.0 per 100,000 inhabitants in 2012 to 916.1 in 2023. On a positive note, in Csongrad-Csanad County, the cancer-related mortality rate dropped from 364.3 per 100,000 in 2012 to 327.9 in 2023, and in Tolna County, deaths from digestive system diseases decreased from 70.6 per 100,000 in 2012 to 65.1 in 2023 [54]. The changes in morbidity and mortality rates present a mixed picture, which can be attributed to the varying impact of HDOs on the population’s health culture, driven by numerous health sociology factors. Additionally, it is a fact that the positive health effects of primary prevention become measurable over several decades. A positive outcome is that in the most vulnerable counties, although not in all indicators, the decline in the population’s health status has been slowed or halted. To accelerate the protective impact of HDOs, a central marketing program would be highly beneficial. It is not enough to establish territorial coverage for HDOs; it is crucial to raise individual awareness of the importance of health promotion. By seeing and internalizing positive models and messages, people can come to value their own health and realize it is worth making efforts to protect it, even in the face of environmental barriers.

Our findings highlight critical disparities in the distribution of Health Development Offices (HDOs) across counties, revealing that while some regions are well-served, others face significant barriers to accessibility. This imbalance poses challenges to equitable access to health development services, particularly for vulnerable groups such as the elderly.

The correlation between county population size and the number of HDOs could prompt decision-makers to reconsider a needs-based distribution, ensuring that regions with higher health burdens and older populations receive adequate support. Additionally, the analysis of various statistical measures, including the Gini Index and entropy, emphasizes the complexity of achieving balanced accessibility across geographic areas.

Overall, our research underscores the importance of aligning the distribution of HDOs with regional health needs, advocating for a more strategic and equitable approach to the spatial placement of critical health development services. As Hungary continues to face the challenges of an aging population, ensuring access to preventive health services through well-structured health development organizations will be vital in promoting healthier aging and reducing regional health disparities.

### Limitations

In addition to the applied statistical methods, it is worth noting the limitations of our research. Our study calculated quantitative concentration but did not examine the quantity and quality of services provided by the offices. Another limitation is that the spatial placement of the Offices could have been influenced by various other health sociological factors, necessitating further analysis. Factors affecting the spatial pattern of Health Development Offices include the economic development of counties, the educational attainment of the population, and other public health indicators.

The low R^2^ coefficient in our initial analysis indicated a weak correlation between population and the number of HDOs. However, after excluding outliers, the R^2^ value improved, showing a stronger relationship. Nevertheless, the linear regression model has limitations in this context. It doesn’t fully capture the impact of other factors like regional healthcare needs or accessibility challenges. We also clarify that the regression was not intended for prediction but was used primarily to visually supplement the Pearson correlation analysis and aid in interpreting the association. Based on the distribution of the scatter plot, a multivariate regression function could provide a better fit, meaning that other factors influenced the territorial placement based on population size, which requires further research.

## Conclusion

The Hungarian Network of Health Development Offices, comprising 108 facilities, effectively meets the preventative healthcare needs of both the general population and individuals over 64 years of age, as indicated by low concentration metrics such as the Lorenz Curve, Gini Index, and Herfindahl–Hirschman Index. Notwithstanding, disparities in the availability of Health Development Offices per 100,000 inhabitants are evident across various counties, including Budapest. This uneven distribution is described by the Location Quotient Index and the values of the entropy index. Therefore, expanding Health Development Offices in underrepresented areas is essential for reducing these disparities and achieving a more balanced county-wide distribution.

The Health Development Offices offer a range of preventative services tailored to the elderly population [11], positioning the network as a key resource for gerontological care in addition to serving the broader active population. Effective coverage, as a key metric for monitoring universal health coverage, highlights the necessity for both service provision and accessibility to ensure equitable health outcomes for all demographics [55]. However, it was the VEKOP grants that facilitated the inclusion of the capital city into the Health Development Offices network, thereby playing a crucial role in establishing a star topology in the distribution of these services where the population was one of the crucial influence factors. The network’s star topology infrastructure is particularly effective in fostering health value attitudes, catering especially well to the elderly.

In the longer term, enhancing the Health Development Office network necessitates deliberate health communication strategies. These strategies should not only aim to expand the network but also to stimulate demand for preventative health services, leveraging the existing infrastructure. Nevertheless, regional development efforts must be supported by further research focused on more effectively enhancing health value attitudes among both the general and elderly populations through strategic adaptations.

Optimizing the Health Development Office network by taking into account the current network topology and spatial distribution is recommended to achieve a decrease in statistical concentration and ensure more equitable coverage. Although the network of Health Development Offices has been established, coverage remains incomplete, with disparities in the availability of offices per 100,000 population across different counties, a finding corroborated by the entropy index values we observed. Future initiatives should prioritize development in regions where the presence of operational Health Development Offices is notably sparse. Crucially, despite intentions to expand, the effectiveness of these offices may be compromised if the absence or inaccessibility of Health Development Offices impedes the utilization of preventive services.

Given their availability, older people are likely to make greater use of these health preventive services. From a gerontological perspective, the physical location and accessibility of these offices are critical, as ease of access in old age is fundamental to service utilisation.

## Supplementary Information

Supplementary Material 1.

## Data Availability

The datasets generated and/or analysed during the current study are available in the Zenodo repository, https://zenodo.org/records/10730741. (DOI: 10.5281/zenodo.10730741). Detailed descriptions of the files uploaded to the Zenodo repository and their sources are available, aiming to enhance transparency and reproducibility. All data generated and analysed during this study are available in our repository, along with a brief statistical summary and any requests, please contact the corresponding author. The Zenodo dataset was updated on 23 September 2024, and the most recent version is available at: 10.5281/zenodo.13826993.

## Abbreviations

H: Shannon Entropy
EFOP: Human Resources Development Operational Programme (EU grant at state member level in Hungary)
G: Gini Index
GIS: Geographic Information System
HDO: Health Development Office
HHI: Herfindahl–Hirschman Index
KSH: Hungarian Central Statistical Office
LQ: Location Quotient Index
MS: Microsoft
NNK: National Public Health Centre
OKFO: National Directorate General for Hospitals
QGIS: Quantum Geographic Information System
R^2^: Coefficient of determination
SPSS: Statistical Package for the Social Sciences
VEKOP: Economic Development and Innovation Operational Programme (EU grant at state member level in Hungary)
