# Supplementary material for: Concentration and geospatial modelling of Health Development Offices’ accessibility for the total and elderly populations in Hungary
Source: BMC Public Health. 2025 Apr 21;25:1466. doi: 10.1186/s12889-025-22392-1 (PMC12010592; doi:10.1186/s12889-025-22392-1)
Supplement: Supplementary file 1 — Supplementary Material 1. [file 12889_2025_22392_MOESM1_ESM.zip › Curve_estimation_elderly_without_outlier.pdf]

Curve Fit

Notes

Output Created		23-SEP-2024 09:53:43
Comments		
Input	Data	C:\PhD\EFI_elérhetőségek\supplementary_files\Send\Data_HDOs_population_without_outliers.sav
	Active Dataset	DataSet1
	Filter	<none>
	Weight	<none>
	Split File	<none>
	N of Rows in Working Data File	18
Missing Value Handling	Definition of Missing	User-defined missing values are treated as missing.
	Cases Used	Cases with a missing value in any variable are not used in the analysis.
Syntax		CURVEFIT<br><br>/VARIABLES=Number_of_HDOs WITH Population_over_64<br>/CONSTANT<br>/MODEL=LINEAR LOGARITHMIC INVERSE QUADRATIC CUBIC COMPOUND POWER S GROWTH EXPONENTIAL LGSTIC<br>/PRINT ANOVA<br>/PLOT FIT.
Resources	Processor Time	00:00:00,19
	Elapsed Time	00:00:00,10
Use	From	First observation
	To	Last observation
Predict	From	First Observation following the use period
	To	Last observation
Time Series Settings (TSET)	Amount of Output	PRINT = DEFAULT
	Saving New Variables	NEWVAR = NONE
	Maximum Number of Lags in Autocorrelation or Partial Autocorrelation Plots	MXAUTO = 16
	Maximum Number of Lags Per Cross-Correlation Plots	MXCROSS = 7

Notes

	Maximum Number of New Variables Generated Per Procedure	MXNEWVAR = 60
	Maximum Number of New Cases Per Procedure	MXPREDICT = 1000
	Treatment of User-Missing Values	MISSING = EXCLUDE
	Confidence Interval Percentage Value	CIN = 95
	Tolerance for Entering Variables in Regression Equations	TOLER = ,0001
	Maximum Iterative Parameter Change	CNVERGE = ,001
	Method of Calculating Std. Errors for Autocorrelations	ACFSE = IND
	Length of Seasonal Period	Unspecified
	Variable Whose Values Label Observations in Plots	Unspecified
	Equations Include	CONSTANT

Warnings

The dependent variable (Number_of_HDOs) contains non-positive values. The minimum value is ,000. Log transform cannot be applied. The Compound, Power, S, Growth, Exponential, and Logistic models cannot be calculated for this variable.

Model Description

Model Name		MOD_2
Dependent Variable	1	Number of HDOs per County without Outliers (capital city and Pest County)
Equation	1	Linear
	2	Logarithmic
	3	Inverse
	4	Quadratic
	5	Cubic
	6	Compound[a]
	7	Power[a]
	8	S[a]
	9	Growth[a]
	10	Exponential[a]
	11	Logistic[a]
Independent Variable		Population over 64 per County without Outliers (capital city and Pest County)
Constant		Included
Variable Whose Values Label Observations in Plots		Unspecified
Tolerance for Entering Terms in Equations		,0001

a. The model requires all non-missing values to be positive.

Case Processing Summary

	N
Total Cases	18
Excluded Cases[a]	0
Forecasted Cases	0
Newly Created Cases	0

a. Cases with a missing value in any variable are excluded from the analysis.

Variable Processing Summary

		Variables	
		Dependent	Independent
		Number of HDOs per County without Outliers (capital city and Pest County)	Population over 64 per County without Outliers (capital city and Pest County)
Number of Positive Values		17	18
Number of Zeros		1[a]	0
Number of Negative Values		0	0
Number of Missing Values	User-Missing	0	0
	System-Missing	0	0

a. The Compound, Power, S, Growth, Exponential, or Logistic model cannot be calculated.

Number of HDOs per County without Outliers (capital city and Pest County)

Linear

Model Summary

R	R Square	Adjusted R Square	Std. Error of the Estimate
,868	,753	,737	1,511

The independent variable is Population over 64 per County without Outliers (capital city and Pest County).

ANOVA

	Sum of Squares	df	Mean Square	F	Sig.
Regression	111,092	1	111,092	48,673	<,001
Residual	36,519	16	2,282		
Total	147,611	17			

The independent variable is Population over 64 per County without Outliers (capital city and Pest County).

Coefficients

	Unstandardized Coefficients		Standardized Coefficients		
	B	Std. Error	Beta	t	Sig.
Population over 64 per County without Outliers (capital city and Pest County)	,000	,000	,868	6,977	<,001
(Constant)	-3,983	1,374		-2,898	,010

Logarithmic

Model Summary

R	R Square	Adjusted R Square	Std. Error of the Estimate
,848	,718	,701	1,612

The independent variable is Population over 64 per County without Outliers (capital city and Pest County).

ANOVA

	Sum of Squares	df	Mean Square	F	Sig.
Regression	106,051	1	106,051	40,829	<,001
Residual	41,560	16	2,597		
Total	147,611	17			

The independent variable is Population over 64 per County without Outliers (capital city and Pest County).

Coefficients

	Unstandardized Coefficients		Standardized Coefficients		
	B	Std. Error	Beta	t	Sig.
ln(Population over 64 per County without Outliers (capital city and Pest County))	8,651	1,354	,848	6,390	<,001
(Constant)	-91,704	15,182		-6,040	<,001

Inverse

Model Summary

R	R Square	Adjusted R Square	Std. Error of the Estimate
,803	,645	,623	1,810

The independent variable is Population over 64 per County without Outliers (capital city and Pest County).

ANOVA

	Sum of Squares	df	Mean Square	F	Sig.
Regression	95,218	1	95,218	29,078	<,001
Residual	52,393	16	3,275		
Total	147,611	17			

The independent variable is Population over 64 per County without Outliers (capital city and Pest County).

Coefficients

	Unstandardized Coefficients B	Unstandardized Coefficients Std. Error	Standardized Coefficients Beta	t	Sig.
1 / Population over 64 per County without Outliers (capital city and Pest County)	-546686,174	101380,728	-,803	-5,392	<,001
(Constant)	12,987	1,492		8,705	<,001

Quadratic

Model Summary

R	R Square	Adjusted R Square	Std. Error of the Estimate
,868	,754	,721	1,556

The independent variable is Population over 64 per County without Outliers (capital city and Pest County).

ANOVA

	Sum of Squares	df	Mean Square	F	Sig.
Regression	111,298	2	55,649	22,987	<,001
Residual	36,313	15	2,421		
Total	147,611	17			

The independent variable is Population over 64 per County without Outliers (capital city and Pest County).

Coefficients

	Unstandardized Coefficients B	Unstandardized Coefficients Std. Error	Standardized Coefficients Beta	t	Sig.
Population over 64 per County without Outliers (capital city and Pest County)	8,826E-5	,000	,634	,782	,446
Population over 64 per County without Outliers (capital city and Pest County) ** 2	2,052E-10	,000	,236	.	.
(Constant)	-2,786	4,344		-,641	,531

Cubic

Model Summary

R	R Square	Adjusted R Square	Std. Error of the Estimate
,870	,757	,705	1,601

The independent variable is Population over 64 per County without Outliers (capital city and Pest County).

ANOVA

	Sum of Squares	df	Mean Square	F	Sig.
Regression	111,735	3	37,245	14,534	<,001
Residual	35,877	14	2,563		
Total	147,611	17			

The independent variable is Population over 64 per County without Outliers (capital city and Pest County).

Coefficients

	Unstandardized Coefficients		Standardized Coefficients	t	Sig.
	B	Std. Error	Beta		
Population over 64 per County without Outliers (capital city and Pest County)	,000	,001	-1,075	-,255	,803
Population over 64 per County without Outliers (capital city and Pest County) ** 2	3,338E-9	,000	3,844	.	.
Population over 64 per County without Outliers (capital city and Pest County) ** 3	-1,299E-14	,000	-1,939	.	.
(Constant)	2,856	14,379		,199	,845

Number of HDOs per County without Outliers (capital city and Pest County)

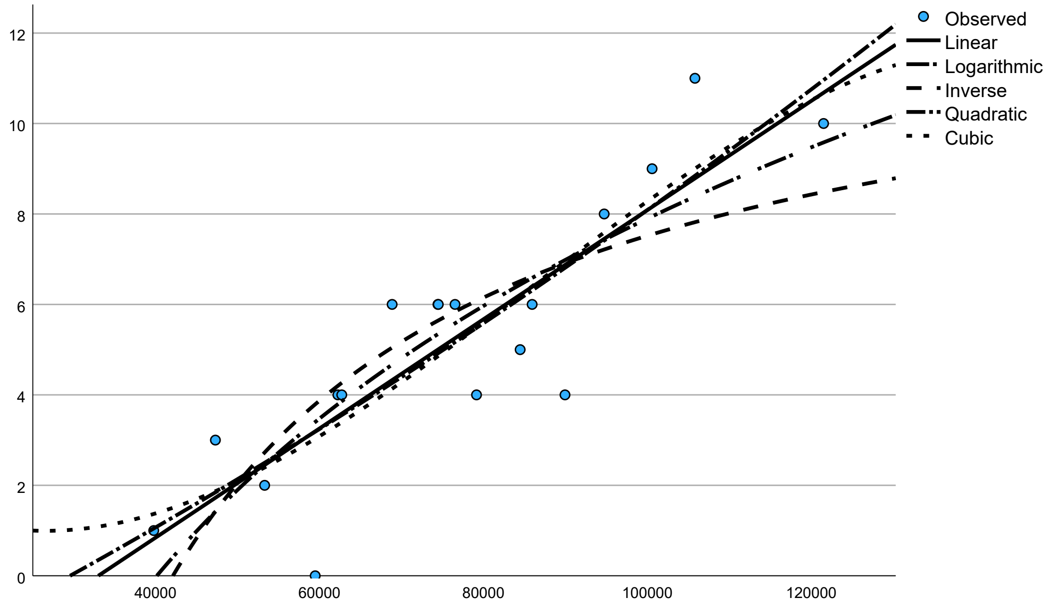

Population over 64 per County without Outliers (capital city and Pest County)
