# Supplementary material for: Concentration and geospatial modelling of Health Development Offices’ accessibility for the total and elderly populations in Hungary
Source: BMC Public Health. 2025 Apr 21;25:1466. doi: 10.1186/s12889-025-22392-1 (PMC12010592; doi:10.1186/s12889-025-22392-1)
Supplement: Supplementary file 1 — Supplementary Material 1. [file 12889_2025_22392_MOESM1_ESM.zip › Curve_estimation_total_population.pdf]

Curve Fit

Notes

Output Created		17-SEP-2024 07:41:19
Comments		
Input	Data	C:<br>\PhD\EFI_elérhetőségek\supplementary_files\SPSS\Data_HDOs_population.sav
	Active Dataset	DataSet0
	Filter	<none>
	Weight	<none>
	Split File	<none>
	N of Rows in Working Data File	20
Missing Value Handling	Definition of Missing	User-defined missing values are treated as missing.
	Cases Used	Cases with a missing value in any variable are not used in the analysis.
Syntax		CURVEFIT<br><br>/VARIABLES=Number_of_HDOs WITH Total_population<br>/CONSTANT<br>/MODEL=LINEAR LOGARITHMIC INVERSE QUADRATIC CUBIC COMPOUND POWER S GROWTH EXPONENTIAL LGSTIC<br>/PRINT ANOVA<br>/PLOT FIT.
Resources	Processor Time	00:00:02,77
	Elapsed Time	00:00:00,50
Use	From	First observation
	To	Last observation
Predict	From	First Observation following the use period
	To	Last observation
Time Series Settings (TSET)	Amount of Output	PRINT = DEFAULT
	Saving New Variables	NEWVAR = NONE
	Maximum Number of Lags in Autocorrelation or Partial Autocorrelation Plots	MXAUTO = 16
	Maximum Number of Lags Per Cross-Correlation Plots	MXCROSS = 7

Notes

	Maximum Number of New Variables Generated Per Procedure	MXNEWVAR = 60
	Maximum Number of New Cases Per Procedure	MXPREDICT = 1000
	Treatment of User-Missing Values	MISSING = EXCLUDE
	Confidence Interval Percentage Value	CIN = 95
	Tolerance for Entering Variables in Regression Equations	TOLER = ,0001
	Maximum Iterative Parameter Change	CNVERGE = ,001
	Method of Calculating Std. Errors for Autocorrelations	ACFSE = IND
	Length of Seasonal Period	Unspecified
	Variable Whose Values Label Observations in Plots	Unspecified
	Equations Include	CONSTANT

```
[DataSet0] C:\PhD\EFI_elérhetőségek\supplementary_files\SPSS\Data_HDOs_population.sav
```

Warnings

The dependent variable (Number_of_HDOs) contains non-positive values. The minimum value is ,000. Log transform cannot be applied. The Compound, Power, S, Growth, Exponential, and Logistic models cannot be calculated for this variable.

Model Description

Model Name		MOD_1
Dependent Variable	1	Number_of_HDOs
Equation	1	Linear
	2	Logarithmic
	3	Inverse
	4	Quadratic
	5	Cubic
	6	Compound[a]
	7	Power[a]
	8	S[a]
	9	Growth[a]
	10	Exponential[a]
	11	Logistic[a]
Independent Variable		Total_population
Constant		Included
Variable Whose Values Label Observations in Plots		Unspecified
Tolerance for Entering Terms in Equations		,0001

a. The model requires all non-missing values to be positive.

Case Processing Summary

	N
Total Cases	20
Excluded Cases[a]	0
Forecasted Cases	0
Newly Created Cases	0

a. Cases with a missing value in any variable are excluded from the analysis.

Variable Processing Summary

		Variables	
		Dependent	Independent
		Number_of_HDOs	Total_population
Number of Positive Values		19	20
Number of Zeros		1[a]	0
Number of Negative Values		0	0
Number of Missing Values	User-Missing	0	0
	System-Missing	0	0

a. The Compound, Power, S, Growth, Exponential, or Logistic model cannot be calculated.

Number_of_HDOs

Linear

Model Summary

R	R Square	Adjusted R Square	Std. Error of the Estimate
,378	,143	,095	2,680

The independent variable is Total_population.

ANOVA

	Sum of Squares	df	Mean Square	F	Sig.
Regression	21,494	1	21,494	2,992	,101
Residual	129,306	18	7,184		
Total	150,800	19			

The independent variable is Total_population.

Coefficients

	Unstandardized Coefficients		Standardized Coefficients		
	B	Std. Error	Beta	t	Sig.
Total_population	2,864E-6	,000	,378	1,730	,101
(Constant)	4,025	,995		4,044	<,001

Logarithmic

Model Summary

R	R Square	Adjusted R Square	Std. Error of the Estimate
,562	,316	,278	2,395

The independent variable is Total_population.

ANOVA

	Sum of Squares	df	Mean Square	F	Sig.
Regression	47,591	1	47,591	8,300	,010
Residual	103,209	18	5,734		
Total	150,800	19			

The independent variable is Total_population.

Coefficients

	Unstandardized Coefficients B	Unstandardized Coefficients Std. Error	Standardized Coefficients Beta	t	Sig.
ln(Total_population)	2,878	,999	,562	2,881	,010
(Constant)	-31,739	12,902		-2,460	,024

Inverse

Model Summary

R	R Square	Adjusted R Square	Std. Error of the Estimate
,679	,462	,432	2,124

The independent variable is Total_population.

ANOVA

	Sum of Squares	df	Mean Square	F	Sig.
Regression	69,598	1	69,598	15,428	<,001
Residual	81,202	18	4,511		
Total	150,800	19			

The independent variable is Total_population.

Coefficients

	Unstandardized Coefficients B	Unstandardized Coefficients Std. Error	Standardized Coefficients Beta	t	Sig.
1 / Total_population	-1566181,819	398742,695	-,679	-3,928	<,001
(Constant)	9,778	1,212		8,070	<,001

Quadratic

Model Summary

R	R Square	Adjusted R Square	Std. Error of the Estimate
,719	,517	,460	2,070

The independent variable is Total_population.

ANOVA

	Sum of Squares	df	Mean Square	F	Sig.
Regression	77,989	2	38,994	9,104	,002
Residual	72,811	17	4,283		
Total	150,800	19			

The independent variable is Total_population.

Coefficients

	Unstandardized Coefficients		Standardized Coefficients	t	Sig.
	B	Std. Error	Beta		
Total_population	2,547E-5	,000	3,358	4,008	<,001
Total_population ** 2	-1,255E-11	,000	-3,042	.	.
(Constant)	-2,290	1,901		-1,205	,245

Cubic

Model Summary

R	R Square	Adjusted R Square	Std. Error of the Estimate
,783	,613	,540	1,910

The independent variable is Total_population.

ANOVA

	Sum of Squares	df	Mean Square	F	Sig.
Regression	92,444	3	30,815	8,449	,001
Residual	58,356	16	3,647		
Total	150,800	19			

The independent variable is Total_population.

Coefficients

	Unstandardized Coefficients		Standardized Coefficients	t	Sig.
	B	Std. Error	Beta		
Total_population	5,103E-5	,000	6,726	3,616	,002
Total_population ** 2	-5,087E-11	,000	-12,330	.	.
Total_population ** 3	1,510E-17	,000	6,034	.	.
(Constant)	-6,890	2,901		-2,375	,030

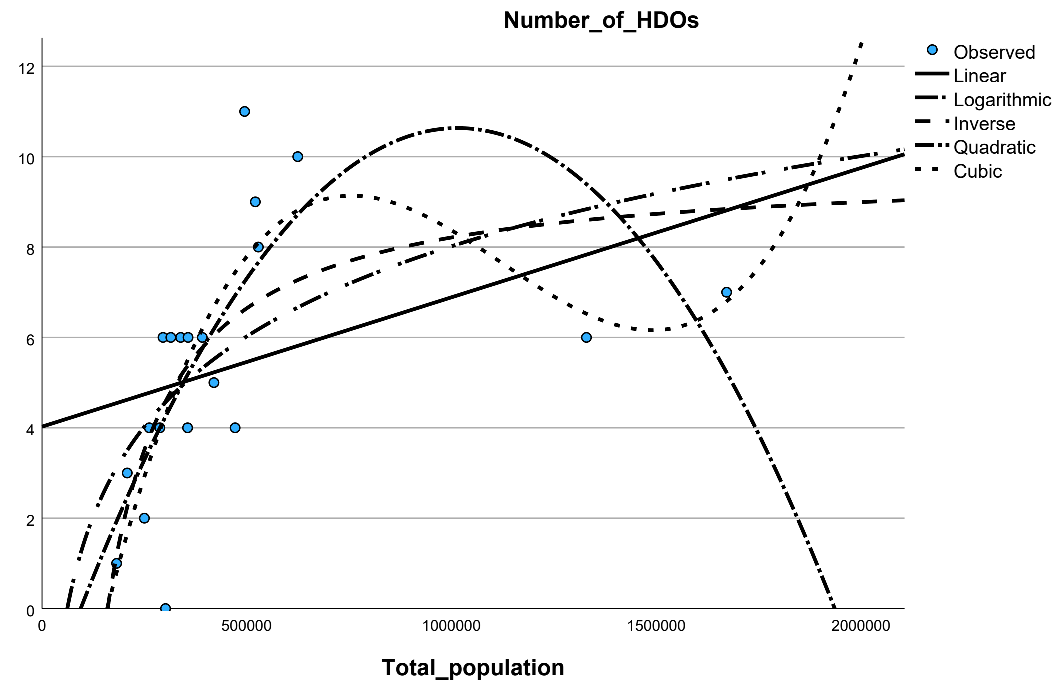
Number_of_HDOs
Observed
Linear
Logarithmic
Inverse
Quadratic
Cubic
0
2
4
6
8
10
12
0
500000
1000000
1500000
2000000
Total_population
