# Supplementary material for: Concentration and geospatial modelling of Health Development Offices’ accessibility for the total and elderly populations in Hungary
Source: BMC Public Health. 2025 Apr 21;25:1466. doi: 10.1186/s12889-025-22392-1 (PMC12010592; doi:10.1186/s12889-025-22392-1)

Curve Fit

Notes

Output Created		17-SEP-2024 08:16:00
Comments		
Input	Data	C:\PhD\EFI_elérhetőségek\supplementary_files\SPSS\Data_HDOs_population.sav
	Active Dataset	DataSet0
	Filter	<none>
	Weight	<none>
	Split File	<none>
	N of Rows in Working Data File	18
Missing Value Handling	Definition of Missing	User-defined missing values are treated as missing.
	Cases Used	Cases with a missing value in any variable are not used in the analysis.
Syntax		CURVEFIT /VARIABLES=Number_of_HDOs WITH Total_population /CONSTANT /MODEL=LINEAR LOGARITHMIC INVERSE QUADRATIC CUBIC COMPOUND POWER S GROWTH EXPONENTIAL LGSTIC /PRINT ANOVA /PLOT FIT.
Resources	Processor Time	00:00:00,31
	Elapsed Time	00:00:00,19
Use	From	First observation
	To	Last observation
Predict	From	First Observation following the use period
	To	Last observation
Time Series Settings (TSET)	Amount of Output	PRINT = DEFAULT
	Saving New Variables	NEWVAR = NONE
	Maximum Number of Lags in Autocorrelation or Partial Autocorrelation Plots	MXAUTO = 16
	Maximum Number of Lags Per Cross-Correlation Plots	MXCROSS = 7

Notes

	Maximum Number of New Variables Generated Per Procedure	MXNEWVAR = 60
	Maximum Number of New Cases Per Procedure	MXPREDICT = 1000
	Treatment of User-Missing Values	MISSING = EXCLUDE
	Confidence Interval Percentage Value	CIN = 95
	Tolerance for Entering Variables in Regression Equations	TOLER = ,0001
	Maximum Iterative Parameter Change	CNVERGE = ,001
	Method of Calculating Std. Errors for Autocorrelations	ACFSE = IND
	Length of Seasonal Period	Unspecified
	Variable Whose Values Label Observations in Plots	Unspecified
	Equations Include	CONSTANT

Model Description

Model Name		MOD_2
Dependent Variable	1	Number_of_HDOs
Equation	1	Linear
	2	Logarithmic
	3	Inverse
	4	Quadratic
	5	Cubic
	6	Compound[a]
	7	Power[a]
	8	S[a]
	9	Growth[a]
	10	Exponential[a]
	11	Logistic[a]
Independent Variable		Total_population
Constant		Included
Variable Whose Values Label Observations in Plots		Unspecified
Tolerance for Entering Terms in Equations		,0001

a. The model requires all non-missing values to be positive.

Case Processing Summary

	N
Total Cases	18
Excluded Cases[a]	0
Forecasted Cases	0
Newly Created Cases	0

a. Cases with a missing value in any variable are excluded from the analysis.

Variable Processing Summary

		Variables	
		Dependent	Independent
		Number_of_HDOs	Total_population
Number of Positive Values		17	18
Number of Zeros		1[a]	0
Number of Negative Values		0	0
Number of Missing Values	User-Missing	0	0
	System-Missing	0	0

a. The Compound, Power, S, Growth, Exponential, or Logistic model cannot be calculated.

Number_of_HDOs

Linear

Model Summary

R	R Square	Adjusted R Square	Std. Error of the Estimate
,797	,635	,612	1,836

The independent variable is Total_population.

ANOVA

	Sum of Squares	df	Mean Square	F	Sig.
Regression	93,699	1	93,699	27,808	<,001
Residual	53,912	16	3,370		
Total	147,611	17			

The independent variable is Total_population.

Coefficients

	Unstandardized Coefficients		Standardized Coefficients		
	B	Std. Error	Beta	t	Sig.
Total_population	1,936E-5	,000	,797	5,273	<,001
(Constant)	-1,820	1,414		-1,287	,216

Logarithmic

Model Summary

R	R Square	Adjusted R Square	Std. Error of the Estimate
,787	,619	,595	1,874

The independent variable is Total_population.

ANOVA

	Sum of Squares	df	Mean Square	F	Sig.
Regression	91,396	1	91,396	26,013	<,001
Residual	56,215	16	3,513		
Total	147,611	17			

The independent variable is Total_population.

Coefficients

	Unstandardized Coefficients		Standardized Coefficients		
	B	Std. Error	Beta	t	Sig.
ln(Total_population)	6,912	1,355	,787	5,100	<,001
(Constant)	-82,916	17,297		-4,794	<,001

Inverse

Model Summary

R	R Square	Adjusted R Square	Std. Error of the Estimate
,752	,566	,539	2,001

The independent variable is Total_population.

ANOVA

	Sum of Squares	df	Mean Square	F	Sig.
Regression	83,541	1	83,541	20,862	<,001
Residual	64,070	16	4,004		
Total	147,611	17			

The independent variable is Total_population.

Coefficients

	Unstandardized Coefficients		Standardized Coefficients	t	Sig.
	B	Std. Error	Beta		
1 / Total_population	-2132320,881	466842,442	-,752	-4,568	<,001
(Constant)	11,741	1,492		7,872	<,001

Quadratic

Model Summary

R	R Square	Adjusted R Square	Std. Error of the Estimate
,797	,635	,586	1,896

The independent variable is Total_population.

ANOVA

	Sum of Squares	df	Mean Square	F	Sig.
Regression	93,707	2	46,853	13,038	<,001
Residual	53,904	15	3,594		
Total	147,611	17			

The independent variable is Total_population.

Coefficients

	Unstandardized Coefficients		Standardized Coefficients	t	Sig.
	B	Std. Error	Beta		
Total_population	2,046E-5	,000	,842	,851	,408
Total_population ** 2	-1,398E-12	,000	-,046	.	.
(Constant)	-2,017	4,488		-,450	,659

Cubic

Model Summary

R	R Square	Adjusted R Square	Std. Error of the Estimate
,797	,635	,557	1,961

The independent variable is Total_population.

ANOVA

	Sum of Squares	df	Mean Square	F	Sig.
Regression	93,752	3	31,251	8,123	,002
Residual	53,859	14	3,847		
Total	147,611	17			

The independent variable is Total_population.

Coefficients

	Unstandardized Coefficients		Standardized Coefficients		
	B	Std. Error	Beta	t	Sig.
Total_population	3,147E-5	,000	1,295	,301	,768
Total_population ** 2	-3,101E-11	,000	-1,020	.	.
Total_population ** 3	2,484E-17	,000	,532	.	.
(Constant)	-3,284	12,580		-,261	,798

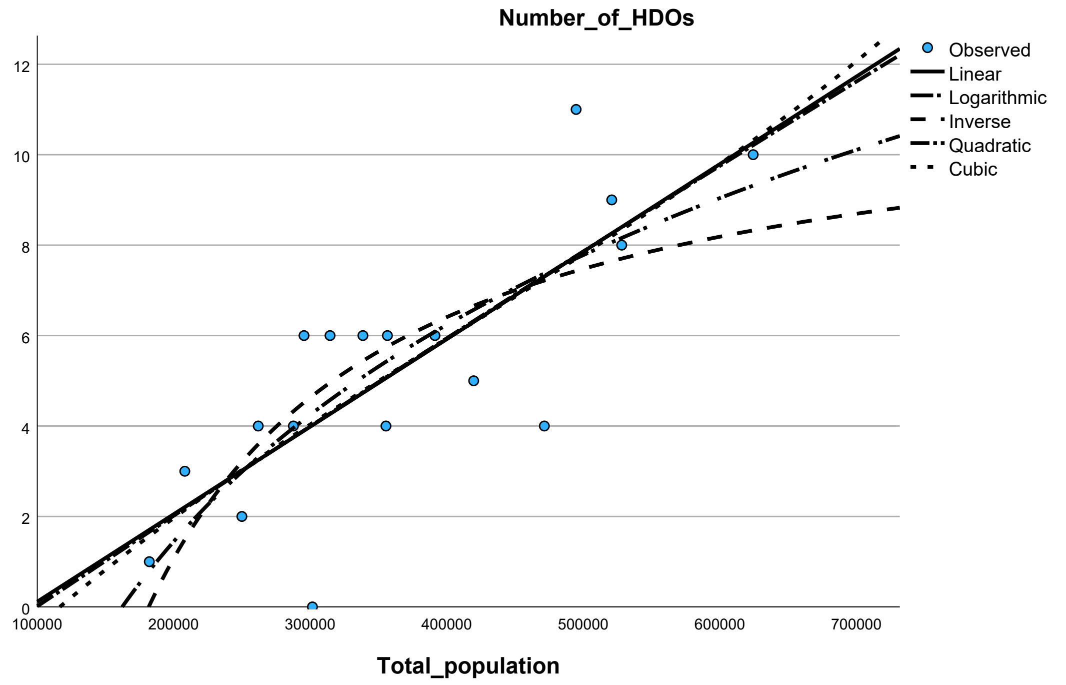

Supplement: Supplementary file 1 — Supplementary Material 1. [file 12889_2025_22392_MOESM1_ESM.zip › Curve_estimation_without_outlier.pdf]
