# Supplementary material for: Concentration and geospatial modelling of Health Development Offices’ accessibility for the total and elderly populations in Hungary
Source: BMC Public Health. 2025 Apr 21;25:1466. doi: 10.1186/s12889-025-22392-1 (PMC12010592; doi:10.1186/s12889-025-22392-1)
Supplement: Supplementary file 1 — Supplementary Material 1. [file 12889_2025_22392_MOESM1_ESM.zip › Dot_HDO_elderly_population.pdf]

Graph

Notes

Output Created		17-SEP-2024 11:21:34
Comments		
Input	Data	C:<br>\PhD\EFI_elérhetőségek\supplementary_files\SPSS\Data_HDOs_population.sav
	Active Dataset	DataSet2
	Filter	<none>
	Weight	<none>
	Split File	<none>
	N of Rows in Working Data File	20
Syntax		GRAPH<br>/SCATTERPLOT(BIVAR)=Population_over_64 WITH Number_of_HDOs<br>/MISSING=LISTWISE<br>/TITLE='The Stochastic Relationship Between the Total Population of the County and the Number '+<br>'of Health Development Offices in Hungary as of 2022 '.
Resources	Processor Time	00:00:00,11
	Elapsed Time	00:00:00,11

```
[DataSet2] C:\PhD\EFI_elérhetőségek\supplementary_files\SPSS\Data_HDOs_population.sav
```

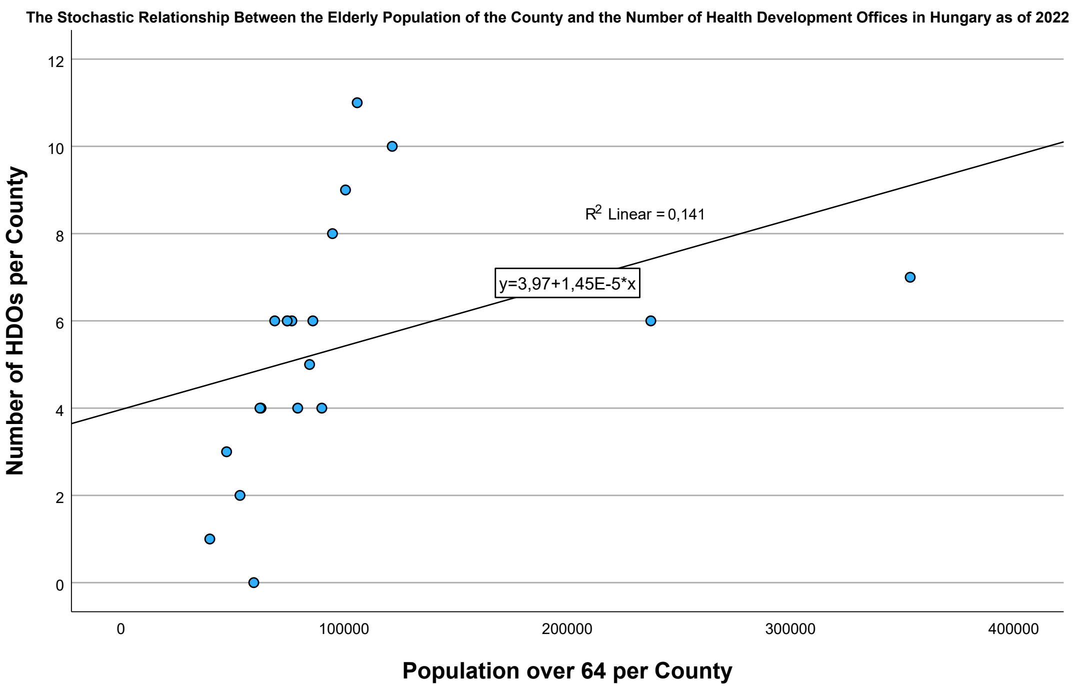

The Stochastic Relationship Between the Elderly Population of the County and the Number of Health Development Offices in Hungary as of 2022
Number of HDOs per County
Population over 64 per County
R2 Linear = 0,141
y=3,97+1,45E-5*x
12
10
8
6
4
2
0
0
100000
200000
300000
400000
