# Supplementary material for: Concentration and geospatial modelling of Health Development Offices’ accessibility for the total and elderly populations in Hungary
Source: BMC Public Health. 2025 Apr 21;25:1466. doi: 10.1186/s12889-025-22392-1 (PMC12010592; doi:10.1186/s12889-025-22392-1)

Graph

Notes

Output Created		17-SEP-2024 09:28:55
Comments		
Input	Data	C: \PhD\EFI_elérhetőségek\supplementary_files\SPSS\Data_HDOs_population.sav
	Active Dataset	DataSet2
	Filter	<none>
	Weight	<none>
	Split File	<none>
	N of Rows in Working Data File	20
Syntax		GRAPH /SCATTERPLOT(BIVAR)=Total_population WITH Number_of_HDOs /MISSING=LISTWISE /TITLE='The Stochastic Relationship Between the Total Population of the County and the Number '+ 'of Health Development Offices in Hungary as of 2022 '.
Resources	Processor Time	00:00:00,13
	Elapsed Time	00:00:00,09

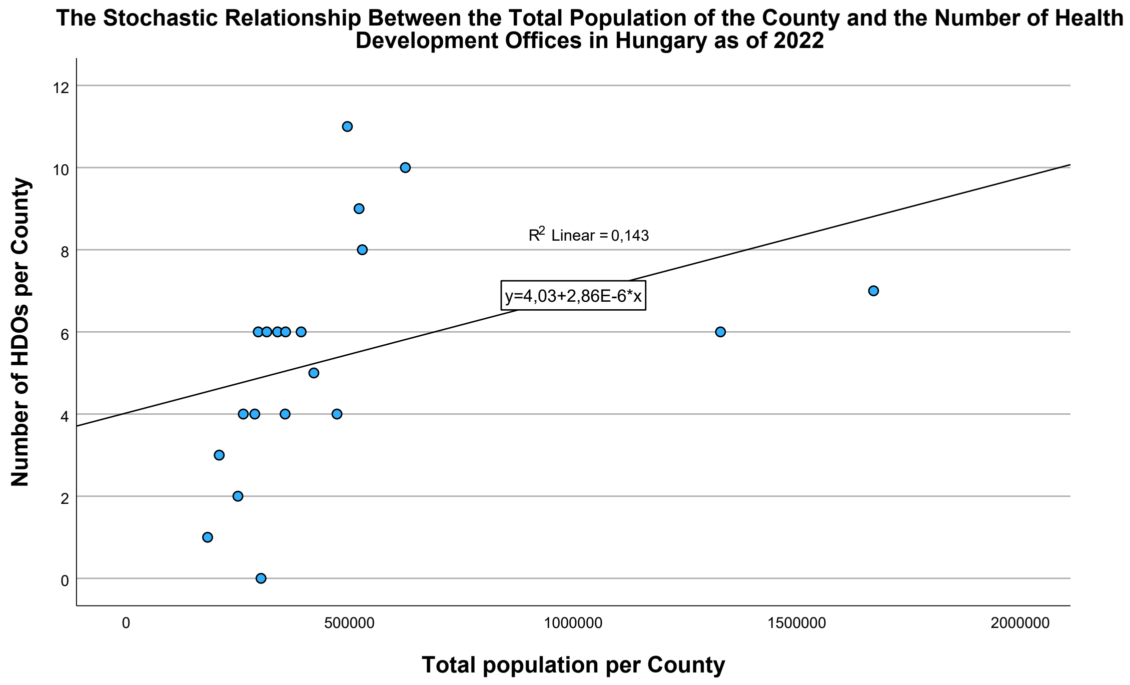

Supplement: Supplementary file 1 — Supplementary Material 1. [file 12889_2025_22392_MOESM1_ESM.zip › Dot_HDO_total_population.pdf]
