# Supplementary material for: Concentration and geospatial modelling of Health Development Offices’ accessibility for the total and elderly populations in Hungary
Source: BMC Public Health. 2025 Apr 21;25:1466. doi: 10.1186/s12889-025-22392-1 (PMC12010592; doi:10.1186/s12889-025-22392-1)
Supplement: Supplementary file 1 — Supplementary Material 1. [file 12889_2025_22392_MOESM1_ESM.zip › Dot_HDO_total_population_without_outliers.pdf]

Graph

Notes

Output Created		17-SEP-2024 09:30:25
Comments		
Input	Data	C:\PhD\EFI_elérhetőségek\supplementary_files\SPSS\Data_HDOs_population_without_outliers.sav
	Active Dataset	DataSet1
	Filter	<none>
	Weight	<none>
	Split File	<none>
	N of Rows in Working Data File	18
Syntax		GRAPH /SCATTERPLOT(BIVAR)=Total_population WITH Number_of_HDOs /MISSING=LISTWISE /TITLE='The Stochastic Relationship Between the Total Population of the County and the Number '+ 'of Health Development Offices in Hungary as of 2022 (without outliers value)'.
Resources	Processor Time	00:00:00,11
	Elapsed Time	00:00:00,08

```
[DataSet1] C:\PhD\EFI_elérhetőségek\supplementary_files\SPSS\Data_HDOs_population_without_outliers.sa
v
```

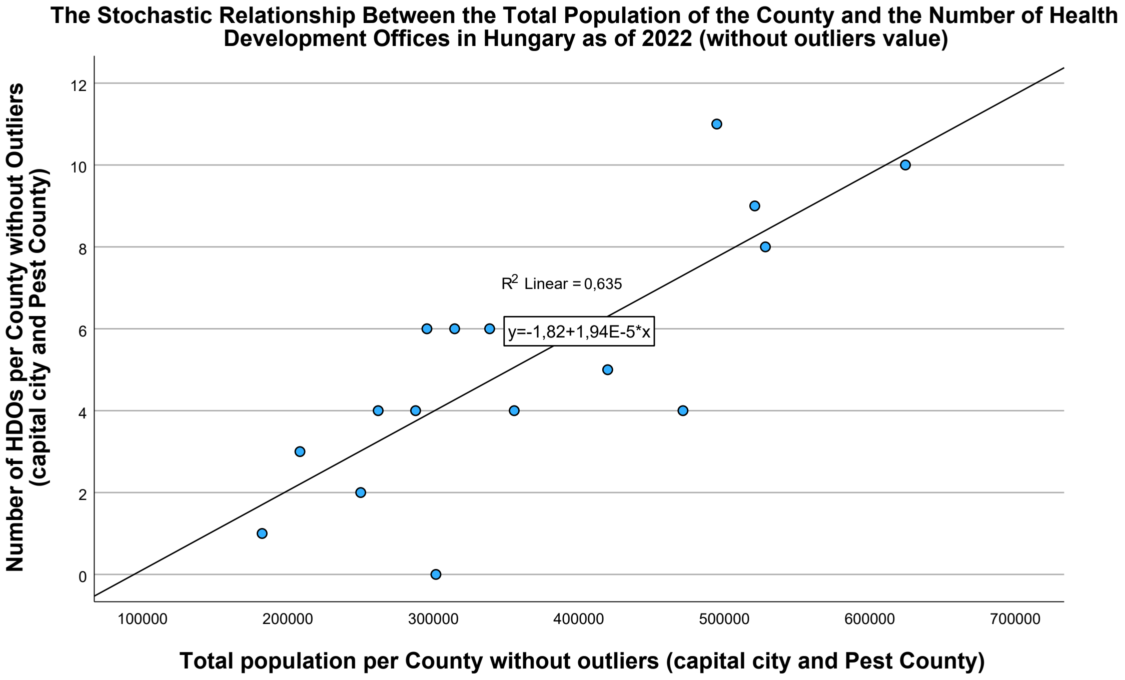
The Stochastic Relationship Between the Total Population of the County and the Number of Health Development Offices in Hungary as of 2022 (without outliers value)
Number of HDOs per County without Outliers (capital city and Pest County)
Total population per County without outliers (capital city and Pest County)
R2 Linear = 0,635
y=-1,82+1,94E-5*x
0
2
4
6
8
10
12
100000
200000
300000
400000
500000
600000
700000
