# Supplementary material for: Concentration and geospatial modelling of Health Development Offices’ accessibility for the total and elderly populations in Hungary
Source: BMC Public Health. 2025 Apr 21;25:1466. doi: 10.1186/s12889-025-22392-1 (PMC12010592; doi:10.1186/s12889-025-22392-1)
Supplement: Supplementary file 1 — Supplementary Material 1. [file 12889_2025_22392_MOESM1_ESM.zip › Pearson_Correlation_populations_HDOs_number.pdf]

Correlations

Notes

Output Created		17-SEP-2024 08:14:26
Comments		
Input	Data	C: \PhD\EFI_elérhetőségek\supplementary_files\SPSS\Data_HDOs_population.sav
	Active Dataset	DataSet0
	Filter	<none>
	Weight	<none>
	Split File	<none>
	N of Rows in Working Data File	20
Missing Value Handling	Definition of Missing	User-defined missing values are treated as missing.
	Cases Used	Statistics for each pair of variables are based on all the cases with valid data for that pair.
Syntax		CORRELATIONS /VARIABLES=Total_population Population_over_64 Number_of_HDOs /PRINT=TWOTAIL NOSIG FULL /MISSING=PAIRWISE.
Resources	Processor Time	00:00:00,05
	Elapsed Time	00:00:00,01

Correlations

		Total_population	Population_over_64	Number_of_HDOs
Total_population	Pearson Correlation	1	,991**	,378
	Sig. (2-tailed)		<,001	,101
	N	20	20	20
Population_over_64	Pearson Correlation	,991**	1	,375
	Sig. (2-tailed)	<,001		,103
	N	20	20	20
Number_of_HDOs	Pearson Correlation	,378	,375	1
	Sig. (2-tailed)	,101	,103	
	N	20	20	20

**. Correlation is significant at the 0.01 level (2-tailed).
