# Supplementary material for: Concentration and geospatial modelling of Health Development Offices’ accessibility for the total and elderly populations in Hungary
Source: BMC Public Health. 2025 Apr 21;25:1466. doi: 10.1186/s12889-025-22392-1 (PMC12010592; doi:10.1186/s12889-025-22392-1)
Supplement: Supplementary file 1 — Supplementary Material 1. [file 12889_2025_22392_MOESM1_ESM.zip › Pearson_Correlation_populations_HDOs_number_without_outliers.pdf]

Correlations

Notes

Output Created		23-SEP-2024 09:49:28
Comments		
Input	Data	C:\PhD\EFI_elérhetőségek\supplementary_files\Send\Data_HDOs_population_without_outliers.sav
	Active Dataset	DataSet1
	Filter	<none>
	Weight	<none>
	Split File	<none>
	N of Rows in Working Data File	18
Missing Value Handling	Definition of Missing	User-defined missing values are treated as missing.
	Cases Used	Statistics for each pair of variables are based on all the cases with valid data for that pair.
Syntax		CORRELATIONS /VARIABLES=Total_population Population_over_64 Number_of_HDOs /PRINT=TWOTAIL NOSIG LOWER /MISSING=PAIRWISE.
Resources	Processor Time	00:00:00,03
	Elapsed Time	00:00:00,01

Correlations

		Total population per County without outliers (capital city and Pest County)	Population over 64 per County without Outliers (capital city and Pest County)	Number of HDOs per County without Outliers (capital city and Pest County)
Total population per County without outliers (capital city and Pest County)	Pearson Correlation	--		
	N	18		
Population over 64 per County without Outliers (capital city and Pest County)	Pearson Correlation	,974**	--	
	Sig. (2-tailed)	<,001		
	N	18	18	
Number of HDOs per County without Outliers (capital city and Pest County)	Pearson Correlation	,797**	,868**	--
	Sig. (2-tailed)	<,001	<,001	
	N	18	18	18

**. Correlation is significant at the 0.01 level (2-tailed).
