# Supplementary material for: Concentration and geospatial modelling of Health Development Offices’ accessibility for the total and elderly populations in Hungary
Source: BMC Public Health. 2025 Apr 21;25:1466. doi: 10.1186/s12889-025-22392-1 (PMC12010592; doi:10.1186/s12889-025-22392-1)
Supplement: Supplementary file 1 — Supplementary Material 1. [file 12889_2025_22392_MOESM1_ESM.zip › Regression_and_outliers_ values.pdf]

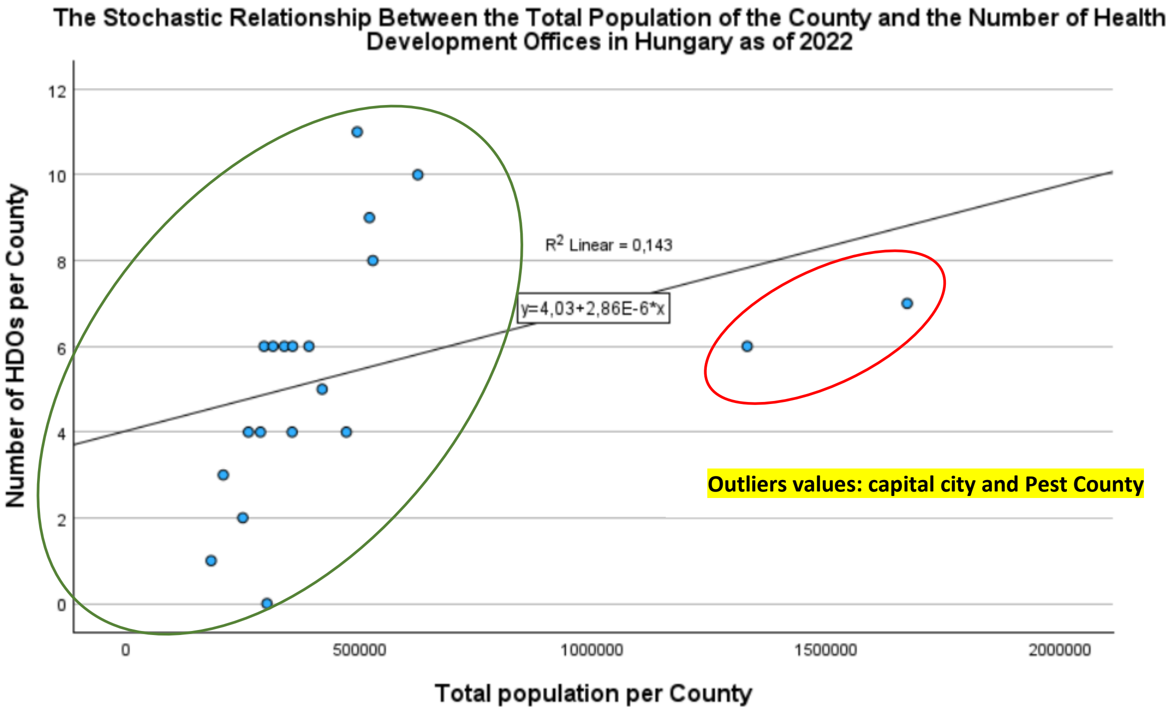
The Stochastic Relationship Between the Total Population of the County and the Number of Health Development Offices in Hungary as of 2022
Number of HDOs per County
Total population per County
R2 Linear = 0,143
y=4,03+2,86E-6*x
Outliers values: capital city and Pest County
0
2
4
6
8
10
12
0
500000
1000000
1500000
2000000

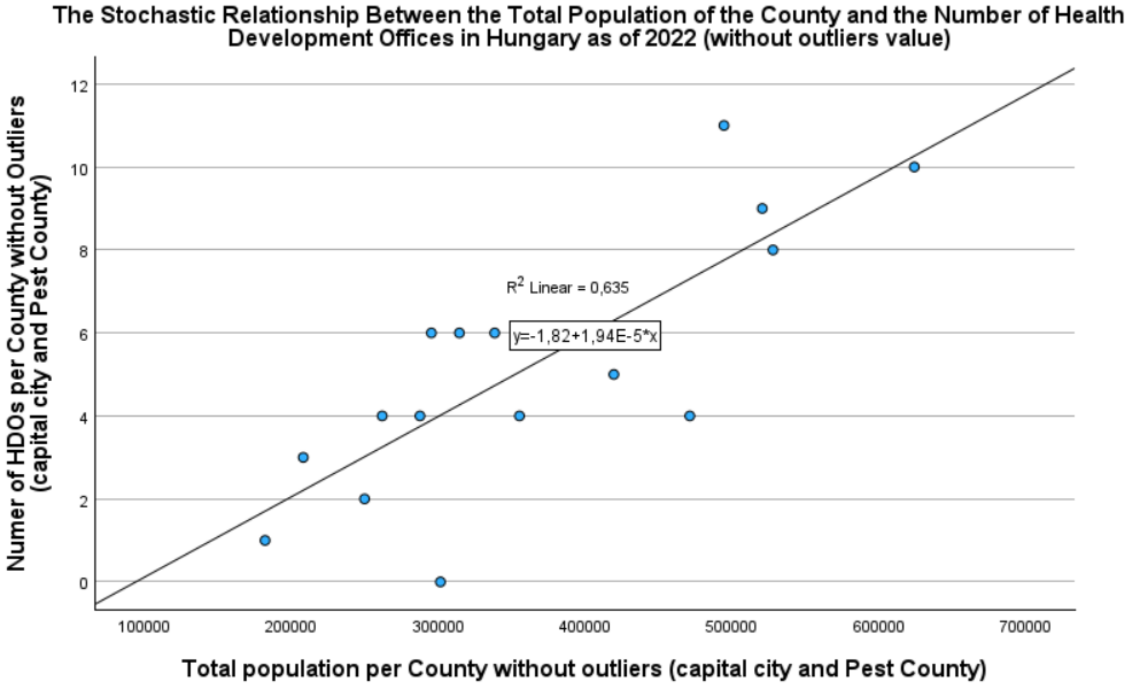
The Stochastic Relationship Between the Total Population of the County and the Number of Health Development Offices in Hungary as of 2022 (without outliers value)
Numer of HDOs per County without Outliers (capital city and Pest County)
Total population per County without outliers (capital city and Pest County)
R2 Linear = 0,635
y=-1,82+1,94E-5*x
0
2
4
6
8
10
12
100000
200000
300000
400000
500000
600000
700000
