# Supplementary material for: Concentration and geospatial modelling of Health Development Offices’ accessibility for the total and elderly populations in Hungary
Source: BMC Public Health. 2025 Apr 21;25:1466. doi: 10.1186/s12889-025-22392-1 (PMC12010592; doi:10.1186/s12889-025-22392-1)
Supplement: Supplementary file 1 — Supplementary Material 1. [file 12889_2025_22392_MOESM1_ESM.zip › Short_Description_of Data_Analysis.pdf]

Short Description of Data Analysis and Attached Files (datasets)

Our research utilised data from 2022, serving as the basis for statistical standardisation. The 2022 Hungarian census provided an objective basis for our analysis, with age group data available at the county level from the Hungarian Central Statistical Office (KSH) website. The 2022 demographic data provided an accurate picture compared to the data available from the 2023 microcensus. The used calculation is based on our standardisation of the 2022 data. For xlsx files, we used MS Excel 2019 (version: 1808, build: 10406.20006) with the SOLVER add-in.

Hungarian Central Statistical Office served as the data source for population by age group, county, and regions: https://www.ksh.hu/stadat_files/nep/hu/nep0035.html, (accessed 04 Jan. 2024.) with data recorded in MS Excel in the *Data_of_demography.xlsx* file.

In 2022, 108 Health Development Offices (HDOs) were operational, and it's noteworthy that no developments have occurred in this area since 2022. The availability of these offices and the demographic data from the Central Statistical Office in Hungary are considered public interest data, freely usable for research purposes without requiring permission.

The contact details for the Health Development Offices were sourced from the following page (Hungarian National Population Centre (NNK)): https://www.nnk.gov.hu/index.php/efi (n=107). The Semmelweis University Health Development Centre was not listed by NNK, hence it was separately recorded as the 108th HDO. More information about the office can be found here: https://semmelweis.hu/egeszsegfejlesztes/en/ (n=1). (accessed 05 Dec. 2023.)

Geocoordinates were determined using Google Maps (N=108): https://www.google.com/maps. (accessed 02 Jan. 2024.) Recording of geocoordinates (latitude and longitude according to WGS 84 standard), address data (postal code, town name, street, and house number), and the name of each HDO was carried out in the: *Geo_coordinates_and_names_of_Hungarian_Health_Development_Offices.csv* file.

The foundational software for geospatial modelling and display (QGIS 3.34), an open-source software, can be downloaded from:
https://qgis.org/en/site/forusers/download.html. (accessed 04 Jan. 2024.)

The HDOs_GeoCoordinates.gpkg QGIS project file contains Hungary's administrative map and the recorded addresses of the HDOs from the *Geo_coordinates_and_names_of_Hungarian_Health_Development_Offices.csv* file, imported via .csv file.

The OpenStreetMap tileset is directly accessible from www.openstreetmap.org in QGIS. (accessed 04 Jan. 2024.)

The Hungarian county administrative boundaries were downloaded from the following website: https://data2.openstreetmap.hu/hatarok/index.php?admin=6 (accessed 04 Jan. 2024.)

HDO_Buffers.gpkg is a QGIS project file that includes the administrative map of Hungary, the county boundaries, as well as the HDO offices and their corresponding buffer zones with a radius of 7.5 km.

Heatmap.gpkg is a QGIS project file that includes the administrative map of Hungary, the county boundaries, as well as the HDO offices and their corresponding heatmap (Kernel Density Estimation).

A brief description of the statistical formulas applied is included in the *Statistical_formulas.pdf.*

Recording of our base data for statistical concentration and diversification measurement was done using MS Excel 2019 (version: 1808, build: 10406.20006) in .xlsx format.

- Aggregated number of HDOs by county: *Number_of_HDOs.xlsx*
- Standardised data (Number of HDOs per 100,000 residents): *Standardized_data.xlsx*
- Calculation of the Lorenz curve: *Lorenz_curve.xlsx*
- Calculation of the Gini index: *Gini_Index.xlsx*
- Calculation of the LQ index: *LQ_Index.xlsx*
- Calculation of the Herfindahl-Hirschman Index: *Herfindahl_Hirschman_Index.xlsx*
- Calculation of the Entropy index: *Entropy_Index.xlsx*
- Regression and correlation analysis calculation: *Regression_correlation.xlsx*

Using the SPSS 29.0.1.0 program, we performed the following statistical calculations with the databases Data_HDOs_population_without_outliers.sav and Data_HDOs_population.sav:

- Regression curve estimation with elderly population and number of HDOs, excluding outlier values (Types of analyzed equations: Linear, Logarithmic, Inverse, Quadratic, Cubic, Compound, Power, S, Growth, Exponential, Logistic, with summary and ANOVA analysis table): Curve_estimation_elderly_without_outlier.spv
- Pearson correlation table between the total population, elderly population, and number of HDOs per county, excluding outlier values such as Budapest and Pest County: Pearson_Correlation_populations_HDOs_number_without_outliers.spv.
- Dot diagram including total population and number of HDOs per county, excluding outlier values such as Budapest and Pest Counties: Dot_HDO_total_population_without_outliers.spv.
- Dot diagram including elderly (64<) population and number of HDOs per county, excluding outlier values such as Budapest and Pest Counties: Dot_HDO_elderly_population_without_outliers.spv
- Regression curve estimation with total population and number of HDOs, excluding outlier values (Types of analyzed equations: Linear, Logarithmic, Inverse, Quadratic, Cubic, Compound, Power, S, Growth, Exponential, Logistic, with summary and ANOVA analysis table): Curve_estimation_without_outlier.spv
- Dot diagram including elderly (64<) population and number of HDOs per county: Dot_HDO_elderly_population.spv
- Dot diagram including total population and number of HDOs per county: Dot_HDO_total_population.spv
- Pearson correlation table between the total population, elderly population, and number of HDOs per county: Pearson_Correlation_populations_HDOs_number.spv
- Regression curve estimation with total population and number of HDOs, (Types of analyzed equations: Linear, Logarithmic, Inverse, Quadratic, Cubic, Compound, Power, S, Growth,

Exponential, Logistic, with summary and ANOVA analysis table): Curve_estimation_total_population.spv

For easier readability, the files have been provided in both SPV and PDF formats.

The translation of these supplementary files into English was completed on 23rd Sept. 2024.
