# Supplementary material for: Concentration and geospatial modelling of Health Development Offices’ accessibility for the total and elderly populations in Hungary
Source: BMC Public Health. 2025 Apr 21;25:1466. doi: 10.1186/s12889-025-22392-1 (PMC12010592; doi:10.1186/s12889-025-22392-1)
Supplement: Supplementary file 1 — Supplementary Material 1. [file 12889_2025_22392_MOESM1_ESM.zip › Statistical_formulas.pdf]

Used Statistical Formulas

Statistical standardization

This process involved using the available county population data and the frequency data of Health Development Offices per county (f(x)) to calculate the indicator of the number of Health Development Offices per 100,000 inhabitants at the level of 2022.

$$\left(\frac{Number\ of\ Health\ Development\ Offices\ at\ the\ county\ level}{Population\ of\ county}\ x\ 100\ 000 \right)$$

Lorenz curve

In this supplementary material, we detail the procedure for constructing the Lorenz curve, a vital tool in our analysis for visualising the distribution of health-related resources across a population. The curve is instrumental in identifying variations in the provision of Health Development Offices per 100,000 individuals within a county (statistical standardization).

Step-by-Step Construction of Lorenz Curve:

Collection of Data: We begin by gathering comprehensive data on the population size and the number of Health Development Offices within the county.

Organisation of Data: Subsequently, we organise the population segments in an ascending order, based on the number of accessible Health Development Offices.

Cumulative Calculations: Cumulative percentages are then computed for both the population and the Health Development Offices, which reflect the proportionate distribution.

Graphical Representation: These cumulative percentages are plotted on a graph, with the population on the horizontal axis and the Health Development Offices on the vertical axis, resulting in a series of points.

Curve Formation: By connecting these points, we generate the Lorenz curve, initiating from the point of origin (0,0) and concluding at the point of complete distribution (100%, 100%) at the Cartesian coordinate system (x,y).

The Lorenz curve's divergence from the line of perfect equality (diagonal line) is indicative of the degree of disparity in distribution. This divergence is quantitatively assessed using the Gini coefficient—a value of 0 denotes absolute equality, whereas a value of 1 expresses maximum inequality.

For our study, we have delineated separate Lorenz curves to compare the overall concentration of Health Development Offices to that accessible to the subset of the population aged above 64. The accompanying Gini coefficients offer a succinct quantification of the distribution inequity within the general and specified elderly population segments.

The Gini index (G) facilitated the validation of our Lorenz curve

$$G = \frac{1}{2n^2\bar{x}} \Sigma_{i=1}^{n} \Sigma_{j=1}^{n} |x_i - x_j|$$

n: number of Hungarian counties
x: number of HDOs per 100,000 population in the examined county (standardized data)

Location Quotient (LQ) Index

$$LQ = \frac{\frac{Number\ of\ HDOs\ in\ an\ examined\ county}{HDOs\ number\ in\ Hungry}}{\frac{The\ population\ of\ the\ examined\ county}{Total\ population\ in\ Hungary}}$$

Herfindahl-Hirschman Index (HHI)

$$HHI = \sum_{i=1}^{n} (Si)^2$$

n: number of counties, S_i: Health Office distribution ratio

Shannon Entropy (E) Index

$$E = -\sum_{i=1}^{n} p_i \times log_{10}(p_i)$$

n: number of counties
p_i relative frequency of Health Development Offices in a selected county

Regression analysis and correlation calculation

In our research, to depict the stochastic relationship between the population size of counties and the number of Health Development Offices, we used a univariate linear correlation equation with MS Excel.

This section shows the statistical approach utilised to examine the stochastic relationship between the county's total population and the number of Health Development Offices.

Analytical Process with MS Excel:

Data Aggregation: Initial data collation involved accumulating the total population figures for each county alongside the respective count of Health Development Offices.

Scatter Plot Generation: We plotted these data points on a scatter plot, with the total population per county on the horizontal axis (X-axis) and the number of Health Development Offices (HDOs) on the vertical axis (Y-axis).

Trend Analysis: To discern the trend, a line of best fit was computed using linear regression, which enabled us to delineate the central tendency amidst the data points.

The regression line is characterised by the equation where y represents the expected number of Health Development Offices and x denotes the total population of the county.

The goodness of fit for the regression model is quantified by the coefficient of determination R^2. This value indicates the proportion of variance in the number of Health Development Offices that can be predicted from the county's population.

References

1. Eshima, N. (2020). Statistical Data Analysis and Entropy, Springer, pp 1-257. doi: https://doi.org/10.1007/978-981-15-2552-0
2. Werden, G. J. (1998). Using the Herfindahl-Hirschman index, Applied Industrial Economics, pp. 368 – 374., doi: https://doi.org/10.1017/CBO9780511522048.021
3. Sitthiyot, T. & Holasut, K. (2021). A simple method for estimating the Lorenz curve. Humanit Soc Sci Commun 8, 268 (2021). https://doi.org/10.1057/s41599-021-00948-x
4. Benassi, F. , Crisci, M. & Rimoldi, S. (2022). Location quotient as a local index of residental segregation. Theoretical and applied aspects. Rivista Italiana di Economia, Demografia e Statistica 76(1) pp. 23-34. https://www.researchgate.net/publication/358462097_Location_quotient_as_a_local_index_of_residential_segregation_Theoretical_and_applied_aspects
5. Walters S., J. (2021). Medical Statistics. Blackwell's, ISBN: 9781119423645, p 448.
